# Binding Modes of Xanthine‐Derived Selective Allosteric Site Inhibitors of MTHFD2

**DOI:** 10.1002/open.202300052

**Published:** 2023-05-02

**Authors:** Vibhu Jha, Leif A. Eriksson

**Affiliations:** ^1^ Department of Chemistry and Molecular Biology University of Gothenburg Medicinaregatan 9c 405 30 Göteborg Sweden

**Keywords:** allosteric site, conformational changes, molecular dynamics simulations, molecular modeling, xanthine-derived MTHFD2 inhibitors

## Abstract

Methylenetetrahydrofolate dehydrogenase (MTHFD2) is a mitochondrial enzyme involved in 1 C metabolism that is upregulated in various cancer cells, but absent in normal proliferating cells. Xanthine derivatives are the first selective inhibitors of MTHFD2 which bind to its allosteric site. Xanthine derivatives (including the co‐crystallized inhibitors) were herein interrogated by molecular/induced‐fit docking, MM‐GBSA binding free energy calculations and molecular dynamics simulations in both MTHFD2 and MTHFD1 (a close homolog expressed in healthy cells). The gained insights from our in silico protocol allowed us to study binding mode, key protein‐ligand interactions and dynamic movement of the allosteric inhibitors, correlating with their experimental binding affinities, biological activities and selectivity for MTHFD2. The reported conformational changes with MTHFD2 upon binding of xanthine derivatives were furthermore evaluated and confirmed by RMSF analyses of the MD simulation trajectories. The results reported herein are expected to benefit in the rational design of selective MTHFD2 allosteric inhibitors.

## Graphical abstract

**Computational modeling of xanthine‐based inhibitor**s binding to the allosteric site of MTHFD2, with promising anticancer activity, was carried out. Using docking/IFD, MD simulations and binding free energy calculations, the binding modes in the MTHFD2 and MTHFD1 allosteric sites were analyzed, establishing a clear correlation with biological activity and selectivity. MD trajectories were furthermore analyzed to verify reported conformational changes in MTHFD2.

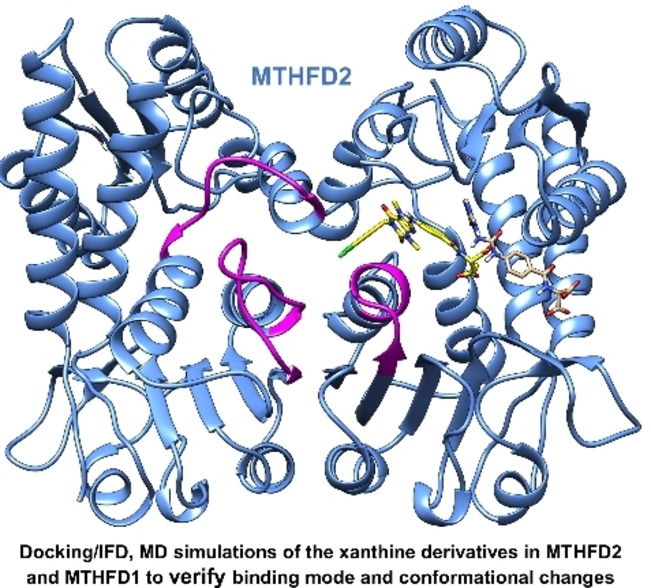

## Introduction

Methylenetetrahydrofolate dehydrogenase/cyclohydrolase (MTHFD2), which plays a key role in 1 C metabolism in purine and thymidine synthesis, is a bifunctional mitochondrial enzyme catalyzing the dehydrogenation of 5,10‐methylene‐THF (CH2−THF) with an NAD^+^ cofactor, and cyclohydrolysis of 5,10‐methenyl‐THF (CH=THF), to yield 10‐formyl‐THF (CHO−THF), eventually producing formate as a 1 C unit.^[1]^ MTHFD2 was found to be overexpressed in various tumors such as breast cancer,^[2]^ colorectal cancer,^[3]^ acute myeloid leukemia^,[4]^,^[5]^ renal cell carcinoma^[6]^ and hepatocellular carcinoma.^[7]^ Upregulation of MTHFD2 may also potentiate the increased risk of bladder cancer.^[8]^ The depletion of MTHFD2 has shown potent tumor suppression effects in the aforementioned cancer cells. MTHFD2 inhibition is supposed to result in increased oxidative stress,^[3]^ glycine dependency,^[9]^ and inadequate purine synthesis^,[10][4]^ among the tumor cells, and suppressing mTORC1 activity through multiple mechanisms which may include guanine depletion and subsequent inhibition of the mTORC1‐activating GTPase Rheb. Furthermore, MTHFD2 is believed to possess non‐metabolic functions involving RNA processing and epigenetic modification^,[11]^,^[12]^ that are essential for cancer cell proliferation.^[13]^ Despite the high expression in various tumors, MTHFD2 is absent/mildly expressed in most of the healthy adult tissues which makes it a prominent target for anticancer drug discovery. The development of MTHFD2 inhibitors could present a new promising therapeutic strategy for MTHFD2‐overexpressing cancers with minimal side effects.^[14]^ Although MTHFD2 is exclusively expressed in cancer cells, a close homolog known as MTHFD1 which shares 36.4 % sequence identity and 53.6 % sequence similarity with MTHFD2, is present in healthy adult tissue,^[15]^ thus raising selectivity concerns in the development of MTHFD2 inhibitors.

**Figure 1:**
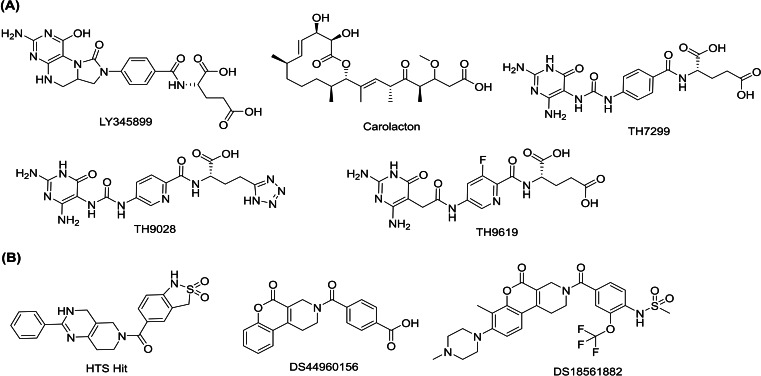
Reported (A) non‐selective and (B) selective inhibitors of MTHFD2.

A few dual MTHFD1/2 inhibitors have been identified in the past. The folate analogue LY345899[^1^, ^14^] (Figure 1A) was found to concurrently inhibit both the isoforms with IC_50_ values of 96 nm and 663 nm for MTHFD1 and MTHFD2, respectively, and giving suppressed tumor growth in a mice xenograft model of colorectal cancer through intraperitoneal injection.^[1]^ Likewise, a natural product known as carolacton (Figure 1A) binds to both MTHFD1 and MTHFD2, showing *K*_i_ values in the nanomolar range.^[16]^ Despite the potent MTHFD2 inhibition, these compounds render a potential safety risk due to the high binding affinity with MTHFD1 which is highly expressed in normal tissues, further substantiating the role of selective MTHFD2 inhibitors for anticancer drug discovery campaigns. Very recently, Bonagas et al.^[5]^ discovered three new MTHFD2 inhibitors ‐ TH7299, TH9028 and TH9619 (Figure 1A) in a high throughput screening (HTS) and structure‐guided lead optimization campaign. Chemically, TH7299, TH9028 and TH9619 are diaminopyrimidine‐based compounds that have shown IC_50_ values of 254 nm, 11 nm and 47 nm, respectively, against MTHFD2 in the biochemical assays. All three inhibitors bind to the substrate binding site of MTHFD2 (PDB codes: 6S4E, 6S4A and 6S4F for TH7299, TH9028 and TH9619, respectively). The diaminopyrimidine‐based inhibitors were also tested in acute myeloid leukemia cells both in vitro and in vivo, and have shown to decrease replication fork speed, further facilitating replication stress and S‐phase arrest. Unfortunately, apart from the nanomolar MTHFD2 inhibitory activity, the three diaminopyrimidine‐based compounds inhibited MTHFD1 and MTHFD2L isoforms in the nanomolar range (MTHFD2L is another isoform expressed in adult tissues^[17]^ and embryonic cells^[18]^). TH7299, TH9028 and TH9619 showed IC_50_ values of 89 nm, 0.5 nm and 16 nm, respectively, against MTHFD1, and 126 nm, 27 nm and 147 nm IC_50_ values, respectively, against MTHFD2L, confirming non‐selective mode of MTHFD2 inhibition.

A tetrahydropyrido[4,3‐d]pyrimidin‐4‐one derivative (HTS hit, Figure 1B, PDB code: 6JID) was the first selective MTHFD2 inhibitor discovered in a HTS campaign using a thermal shift assay. The HTS hit showed a moderately potent inhibition of MTHFD2 with an IC_50_ value of 8.3 μm, however, strikingly did not show any activity against MTHFD1 isoform (IC_50_>100 μm).^[19]^ Rational structure‐based drug design of the HTS hit led to the discovery of lead compound DS44960156 (Figure 1B, PDB code: 6JIB), which is a tricyclic coumarin‐based derivative, showing an IC_50_ of 1.6 μm and >18‐fold selectivity for MTHFD2. The best compound from the ‘DS’ series was reported as DS18561882 (Figure 1B), which is an orally available MTHFD2 inhibitor, exhibiting 0.0063 μm and 0.57 μm IC_50_ values for MTHFD2 and MTHFD1, respectively. DS18561882 furthermore displayed a decent pharmacokinetic profile and high cell‐based activity against the MDA‐MB‐231 cell line derived from human breast cancer, with a GI_50_ of 0.14 μm. ^[20]^

**Figure 2:**
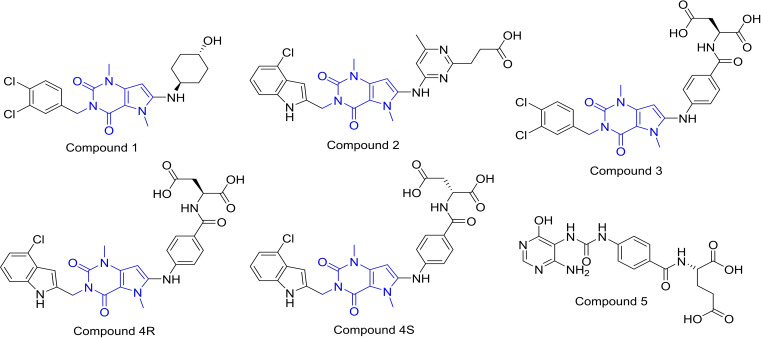
Co‐crystallized/reported MTHFD2 allosteric inhibitors (compounds **1**–**3**, **4 R** and **4 S**) and the folate‐based inhibitor (compound **5**).

Xanthine derivatives which bind selectivity to MTHFD2, were discovered by Lee et al.^[21]^ Instead of the substrate binding site, the xanthine derivatives occupy an allosteric site of MTHFD2, precluding the binding of the cofactor and phosphate to MTHFD2. MTHFD2 is a homodimeric protein in which xanthine‐based inhibitors bind to both the dimers (for clarity, the binding mode of xanthine derivatives in this work are shown with respect to binding with monomer A only). Xanthine derivatives interact with the monomer A of MTHFD2; however, a few residues from monomer B (such as Leu167) are in close proximity to the inhibitors. Three X‐ray structures of MTHFD2 in complex with xanthine derivatives were determined. Each three of them composed of a selective allosteric inhibitor (Figure 2); compound **1** (PDB code: 7EHV), compound **2** (PDB code: 7EHN), and compound **3** (PDB code: 7EHM), and coexisting with a folate‐based inhibitor (compound **5**, which resembles tetrahydrofolate). The ternary X‐ray structures confirmed that xanthine derivatives were bound to the allosteric site, while compound **5** occupied the substrate binding site of MTHFD2. Additionally, another X‐ray structure of MTHFD2 without the allosteric inhibitor, however, in complex with the folate‐based compound **5**, cofactor NAD^+^ and pyrophosphate (P_i_) was solved (PDB code: 7EHJ).

**Figure 3:**
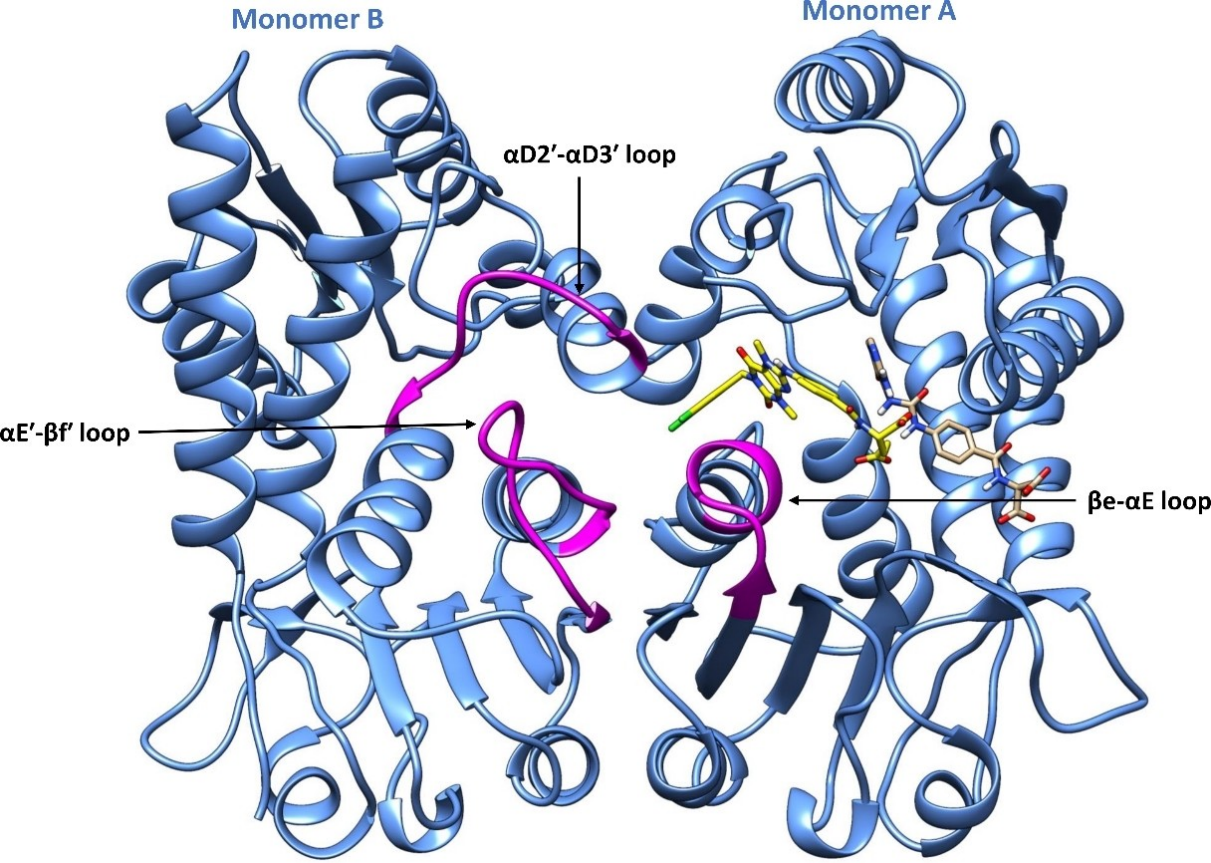
Reported conformational changes of the βe‐αE loop (magenta, monomer A), the αD2′‐αD3′ loop (magenta, monomer B) and the αE′‐βf′ loop (magenta, monomer B) in the presence of the xanthine‐based inhibitor (compound **3**, in yellow) at the MTHFD2 allosteric site. Protein ribbons are colored in blue, and the folate‐based inhibitor (compound **5**) in tan, positioned in the MTHFD2 substrate binding site (PDB code: 7EHM).

On comparing and superposing the four above‐mentioned X‐ray crystal structures (three structures containing an allosteric inhibitor and one structure lacking this), it was found that MTHFD2 went through various conformational changes when bound to the allosteric inhibitors, consequently impeding the binding of the cofactor and phosphate to MTHFD2. In particular, the βe‐αE loop (monomer A), the αD2′‐αD3′ loop (monomer B) and the αE′‐βf′ loop (monomer B) tend to move away from the allosteric site of MTHFD2 when the xanthine‐based inhibitors are bound (Figure 3), while in absence of the allosteric inhibitors (compound **5**, PDB code: 7EHJ), no conformational changes were detected. Furthermore, kinetic studies on MTHFD2 inhibition by compounds **1–3** were performed in order to explicate the mechanism of the enzymatic inhibition. The extent of MTHFD2 inhibition at any fixed concentration of the folate‐based inhibitor decreased on increasing the substrate concentration (tetrahydrofolate), which underlines that compound **5** underwent a competitive mode of MTHFD2 inhibition.^[21]^ In contrary to this, the xanthine derivatives demonstrated an entirely different response relative to the folate‐based inhibitor. The extent of MTHFD2 inhibitory activity increased with increasing substrate concentration at a given concentration of compounds **2** and **3**, demonstrating MTHFD2 inhibition in a non‐competitive manner. The outcomes from the kinetics studies of compounds **1**–**3** and compound **5** on MTHFD2 inhibition thus establish a good correlation with the aforementioned X‐ray structures, confirming a new and allosteric binding mode of xanthine derivatives, and featuring formation of catalytically inactive ternary complexes (enzyme‐substrate‐inhibitor).^[21]^ To the best of our knowledge, the only selective inhibitors of MTHFD2 are the tricyclic coumarin‐based compounds identified by Kawai et al.[^19^, ^20^] and the xanthine‐based compounds developed by Lee et al.^[21]^

In the present work, we exclusively focused on the allosteric site of MTHFD2. The X‐ray structures of MTHFD2 in complex with the xanthine‐based allosteric inhibitors (compounds **1**–**3**) were analyzed by molecular dynamics (MD) simulations. The crystallographic binding mode, conformational dynamics and protein‐ligand interactions of compounds **1**–**3** were thoroughly investigated to explore the correlation with potent biological activity and selectivity for MTHFD2. In addition, we performed computational modeling of the most potent inhibitor; compound **4** (both enantiomers, **4 R** and **4 S**), employing molecular docking and MD simulations since no previous crystallographic or docking data was available. Docking and MD simulations guided us to determine the putative binding modes of compounds **4 R** and **4 S**, correlating with the experimental binding affinity of the preferred enantiomer. The reported conformational changes induced by compounds **1**–**3**, **4 R** and **4 S** at the allosteric site of MTHFD2 were verified from the MD trajectories and the corresponding RMSF analysis. Furthermore, as the above‐mentioned allosteric inhibitors of MTHFD2 (compounds **1**–**3**, **4 R** and **4 S**) are reported to be selective, we performed docking, induced‐fit docking and MD simulations on the co‐crystallized structure of MTHFD1 (PDB code: 6ECQ) to gain deeper insights on the cause of the poor binding to MTHFD1, influencing MTHFD2 selectivity. The selectivity preference of all compounds for MTHFD2 over MTHFD1 as suggested by the co‐crystallized/docking poses and MD simulations was further examined by MM‐GBSA binding free energy calculations on both the MTHFD2 and MTHFD1 isoforms.

## Results and Discussion

### Availability of X‐ray crystal structures and published inhibitors

Compound **1** (PDB code: 7EHV), compound **2** (PDB code: 7EHN) and compound **3** (PDB code: 7EHM) were co‐crystallized with MTHFD2 and each of them co‐exists with the folate‐based inhibitor (compound **5**, in the substrate binding site) in the form of ternary complexes. The IC_50_ values of compound **1**, **2** and **3** were found to be 4.0 μm, 0.69 μm and 0.78 μm, respectively, for MTHFD2.^[21]^ An X‐ray structure of MTHFD2 is also co‐crystallized with the folate‐based inhibitor (compound **5** in the substrate binding site) in the absence of an allosteric inhibitor (PDB code: 7EHJ). Compound **4 R** (Figure 2) was reported to be the most potent inhibitor among the xanthine derivatives with an IC_50_ value of 0.19 μm; however, no crystallographic or modeling data was available. Apart from the allosteric inhibitors, a few substrate site inhibitors have also been co‐crystallized; HTS Hit (PDB code: 6JID) and DS44960156 (PDB code: 6JIB) which bind selectively to the MTHFD2 isoform.[^19^, ^20^]

### Comparison of MTHFD1 and MTHFD2 proteins

**Figure 4:**
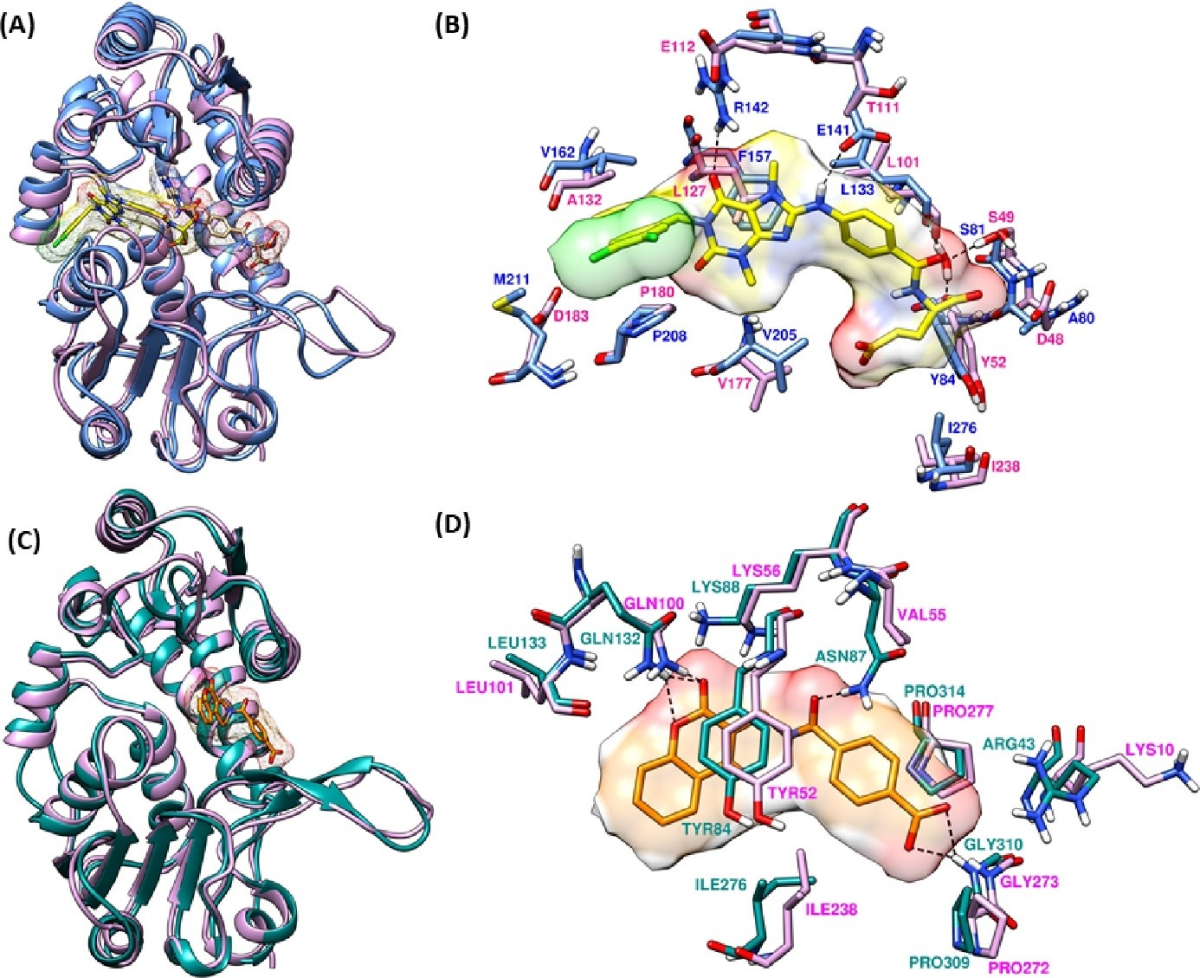
(A) X‐ray structure of MTHFD2 (PDB code: 7EHM, monomer A, blue ribbons) superposed with the X‐ray structure of MTHFD1 (PDB code: 6ECQ, monomer A, pink ribbons). Xanthine‐based compound **3** (yellow) at the allosteric site, folate‐based compound **5** (tan) at the substrate binding site of MTHFD2. (B) Allosteric site residues are superposed and labelled (blue – MTHFD2, pink – MTHFD1). Compound **3** is shown in yellow. (C) X‐ray structure of MTHFD2 (PDB code: 6JIB, monomer A, cyan ribbons) superposed with the X‐ray structure of MTHFD1 (PDB code: 6ECQ, monomer A, pink ribbons). Tricyclic coumarin‐based inhibitor DS44960156 (orange) at the substrate site of MTHFD2. (D) Substrate site residues are superposed and labelled (cyan – MTHFD2, pink – MTHFD1). DS44960156 is shown in orange.

As discussed in the introduction, MTHFD2 is exclusively expressed in cancer cells whereas its close homolog MTHFD1 is present in healthy adult tissue.^[15]^ MTHFD1 shares 36.4 % sequence identity and 53.6 % sequence similarity with MTHFD2; thus, the development of new and potent inhibitors binding to MTHFD2, selectively with no or poor binding on MTHFD1, is of great importance. In order to gain deeper insight on the allosteric site and the selectivity preference, we thus superposed the X‐ray structure of MTHFD2 in complex with compound **3** (PDB code: 7EHM) and the X‐ray structure of MTHFD1 in complex with the folate‐based inhibitor (PDB code: 6ECQ). Figures 4A and 4B depict the superposition between the allosteric site of MTHFD2 (bound to compound **3**) and the allosteric site of MTHFD1. The amino linker of compound **3** engages in an H‐bond interaction with the Glu141 sidechain, while one of the carbonyl groups from the central xanthine unit of compound **3** forms a H‐bond with the sidechain of Arg142. Furthermore, the purine core establishes π–π stacking with Phe157. These three protein‐ligand interactions between compound **3** and the MTHFD2 allosteric site shed some light on selectivity; Glu141 in MTHFD2 is replaced by Thr111 in MTHFD1, that is inaccessible to the amino linker of compound **3**. Similarly, Glu112 in MTHFD1 replaces Arg142 in MTHFD2, and is projected away from the central xanthine core of compound **3**, resulting in loss of H‐bond contact. Finally, substitution of Phe157 in MTHFD2 with Leu127 in MTHFD1 impairs the π–π interactions with the purine moiety of the xanthine core, facilitating the selectivity for MTHFD2. In order to develop selective MTHFD2 inhibitors, the presence of H‐bond interactions with Glu141 and Arg142, and lipophilic contact with Phe157 at the allosteric site of MTHFD2 seem to be integral elements that must be met by the potential compounds resulting from the structure‐guided development/optimization.

Similarly, to shed light on selectivity inclination at the substrate site level, the X‐ray structure of MTHFD2 in complex with DS44960156 (PDB code: 6JIB) was superposed with the X‐ray structure of MTHFD1 in complex with the folate‐based inhibitor (PDB code: 6ECQ, the co‐crystallized folate‐based inhibitor of MTHFD1 is hidden for clarity, however the full binding site view of MTHFD1 – folate‐based inhibitor is shown in Figure S20). As shown in Figures 4C and 4D, significant H‐bond interactions were noted between the sidechains of Asn87, Lys88 and Gln132, and inhibitor DS44960156. Furthermore, DS44960156 establishes H‐bond contact with the backbone nitrogen of Gly310, and a π–π stacking with Tyr84. These protein‐ligand interactions are commonly observed with the substrate site inhibitors of MTHFD2 (HTS Hit and DS18561882), contributing to the binding affinity for MTHFD2 and thus considered as key interactions. In addition, MD simulation analysis from our previous studies^[22]^ revealed that DS44960156 forms notable H‐bond contact with Arg43 despite that this interaction was not seen in the crystallographic pose. Tyr84, Lys88, Gln132 and Gly310 in MTHFD2 are identically replaced by Tyr52, Lys56, Gln100 and Gly273 in MTHFD1, respectively, whereas Arg43 and Asn87 are replaced by Lys10 and Val55, respectively. The H‐bond interaction between the inhibitor (DS44960156) and the hydrophilic Asn87 of MTHFD2 is crucial to govern selectivity as MTHFD1 has the hydrophobic Val55 in the same position, which is placed slightly away from the substrate binding site. Moreover, despite being structurally homologous, Lys10 in MTHFD1 is projected toward the solvent‐exposed region while Arg43 at the same position in MTHFD2 is oriented next to Gly310 within the substrate binding site, thus offering more opportunities for the inhibitor to extend the H‐bond network and provide selectivity for MTHFD2. Altogether, the MTHFD2 allosteric site residues Gl141, Arg142 and Phe157 that are replaced by Thr111, Glu112 and Leu127 in MTHFD1, respectively, and the MTHFD2 substrate site residues Arg43 and Asn87 that are replaced by Lys10 and Val55, respectively, are considered vital for facilitating selective MTHFD2 inhibition, thereby offering significant protein‐ligand interactions to be exploited by novel ligands for improving MTHFD2 potency and selectivity.

Due to the limited structural availability of existing inhibitors in complex with MTHFD1/MTHFD2, the current study is completely done in silico and exclusively aimed at the structural interrogation of xanthine‐derived allosteric inhibitors of MTHFD2 (compounds **1**–**3**, **4 R** and **4 S**) by molecular docking, molecular dynamics simulations and MM‐GBSA binding free energy calculations. The computational protocol also allowed us to validate the poor binding of these inhibitors in MTHFD1, correlating with the experimental results and further confirming selective MTHFD2 inhibition. The key findings of this work involving essential protein‐ligand interactions, desirable binding modes, conformational changes at the allosteric site of MTHFD2 as well as poor binding poses with MTHFD1, are expected to guide in the rational structure‐based design of new, selective and potent allosteric inhibitors of MTHFD2.

### MD simulations of the MTHFD2 co‐crystallized inhibitors

With the purpose of evaluating the dynamic movement, the stability of the binding mode and the existence of protein‐ligand interactions, the co‐crystallized allosteric inhibitors of MTHFD2 (compounds **1**–**3**) were subjected to 200 ns MD simulations starting from their crystallographic poses. As a measure of ligand mobility, the Root Mean Square Deviations (RMSD) of the ligand heavy atoms were calculated throughout the simulations. The RMSD represents the deviation of the atoms from their initial crystallographic/suggested binding pose during the simulation. Similarly, the RMSDs of the protein α‐carbons were calculated over the course of the simulations, as a measure of protein mobility.

**Figure 5:**
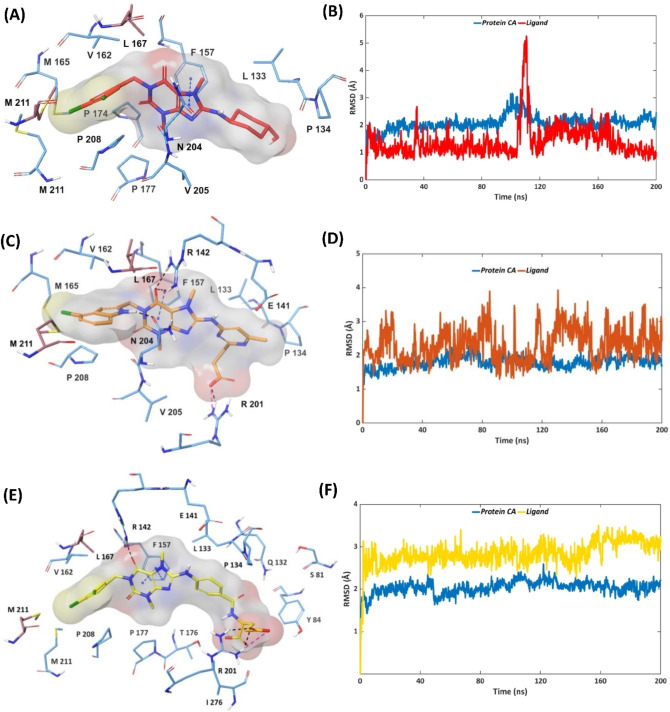
Representative MD structures of MTHFD2 ‐ inhibitor complexes (left column) and RMSD analyses during 200 ns simulations (right column), protein α‐carbons in blue. (A, B) MTHFD2 ‐ compound **1**; (C, D) MTHFD2 ‐ compound **2**; (E, F) MTHFD2 ‐ compound **3**.

As evident from the crystallographic pose (Figure S1), the central purine scaffold of compound **1** establishes π–π stacking with Phe157 in the MTHFD2 allosteric site. Furthermore, the dichlorophenyl ring of compound **1** forms van der Waals contacts with the Val162 sidechain. Pro208 of the MTHFD2 allosteric site was found to be in proximity of compound **1**, thus contributing via additional lipophilic contacts. The stability of the binding mode of compound **1** was confirmed by MD simulations, highlighting the existence of the interactions with Phe157, Val162, and Pro208 for 100 %, 52 % and 34 % of the simulation time, respectively (Figures 5A, 5B, S8). No H‐bond contacts were observed between compound **1** and the allosteric site, correlating with its mid‐micromolar biological activity (IC_50_=4.0 μm) against MTHFD2. Furthermore, compound **1** exhibits great stability throughout the simulation, albeit with a noteworthy fluctuation between 105–114 ns of the simulation trajectory. The RMSD fluctuation corresponds to the dynamic movement and conformational changes of the solvent‐exposed cyclohexanol unit of compound **1**. Compound **2** retains π–π stacking with Phe157 in the MTHFD2 allosteric site. The dichlorophenyl ring in compound **1** is substituted by a 4‐chloroindole ring system in compound **2**, which is sandwiched between Val162 and Pro208, establishing lipophilic interactions. Furthermore, the amino group of this 4‐chloroindole unit forms a H‐bond contact with Asn204. (Figure S2). MD analysis of compound **2** revealed that the interactions with Phe157, Val162 and Pro208 account for 100 %, 36 % and 21 % of the simulation trajectory, respectively. The H‐bond contact between the 4‐chloroindole of compound 2 and Asn204 was furthermore maintained throughout the simulation, and the two arginine residues in the MTHFD2 allosteric site, Arg142 and Arg201, were found to interact abundantly with compound **2** as observed from the MD analysis. The Arg142 sidechain H‐bonds to one of the carbonyl groups of the xanthine core of compound **2** throughout the simulation time. The Arg201 sidechain contributes via H‐bond contact (90 %) and salt‐bridge interaction (11 %) with the carboxylate of the propanoic acid moiety, and lipophilic contact (36 %) with the 6‐methyl pyrimidine ring, respectively, during the course of the simulation (Figures 5C, 5D, S9). Thus, apart from the lipophilic contacts, the presence of new and remarkably stable H‐bond interactions (with Arg142, Arg201 and Asn204) play a significant role in improving the binding affinity and the corresponding MTHFD2 inhibition of compound **2** (IC_50_=0.69 μm). Furthermore, compound **2** exhibits a reasonable stability throughout the simulation without showing any notable RMSD fluctuations.

As apparent from the crystallographic pose, compound **3** provides lipophilic contacts to Phe157, Val162 and Pro208 in the MTHFD2 allosteric site, similar to compounds **1** and **2**. One of the carbonyl groups from the xanthine core of compound **3** forms an H‐bond interaction with the Arg142 sidechain. Furthermore, the amino linker connecting the xanthine core and the phenyl ring of compound **3** is anchored by an H‐bond contact with the Glu141 sidechain. The long glutamic acid unit of compound **3** forms water‐mediated H‐bond contacts with Ser81, Gln132 and Leu133 which are part of the MTHFD2 substrate binding site (Figure S3). As observed from the MD analysis, the lipophilic contacts between compound **3** and Phe157, Val162 and Pro208 exist for 100 %, 60 % and 17 % of the simulation time, respectively (Figures 5E, 5F, S10). The H‐bond contacts observed between compound **2** and Arg142 and Arg201 were maintained abundantly also for compound **3** for 98 % and 100 % of the simulation trajectory, respectively. A couple of new H‐bond contacts were detected at the oxygen backbone of the compound 3 glutamic acid moiety, with the solvent‐exposed Arg278 sidechain for 48 % of the simulation, and with the Tyr84 sidechain for 40 % of the simulation. In addition, the phenyl ring of compound **3** forms van der Waals contacts with Leu133 for 33 % of the simulation time whereas the 4‐chloroindole ring system further facilitates lipophilic interactions with Val167 (monomer B) for 32 % of the simulation trajectory. The presence of extensive H‐bond interactions and lipophilic contacts between compound 3 (particularly due to the long glutamic acid) and MTHFD2 are believed to potentiate the binding affinity and MTHFD2 inhibition, corresponding to an IC_50_ value of 0.78 μm. Moreover, compound **3** exhibits significant stability in terms of ligand mobility, as evident from the RMSD plot.

### Molecular docking and MD simulations of compounds 4 R and 4 S

**Figure 6:**
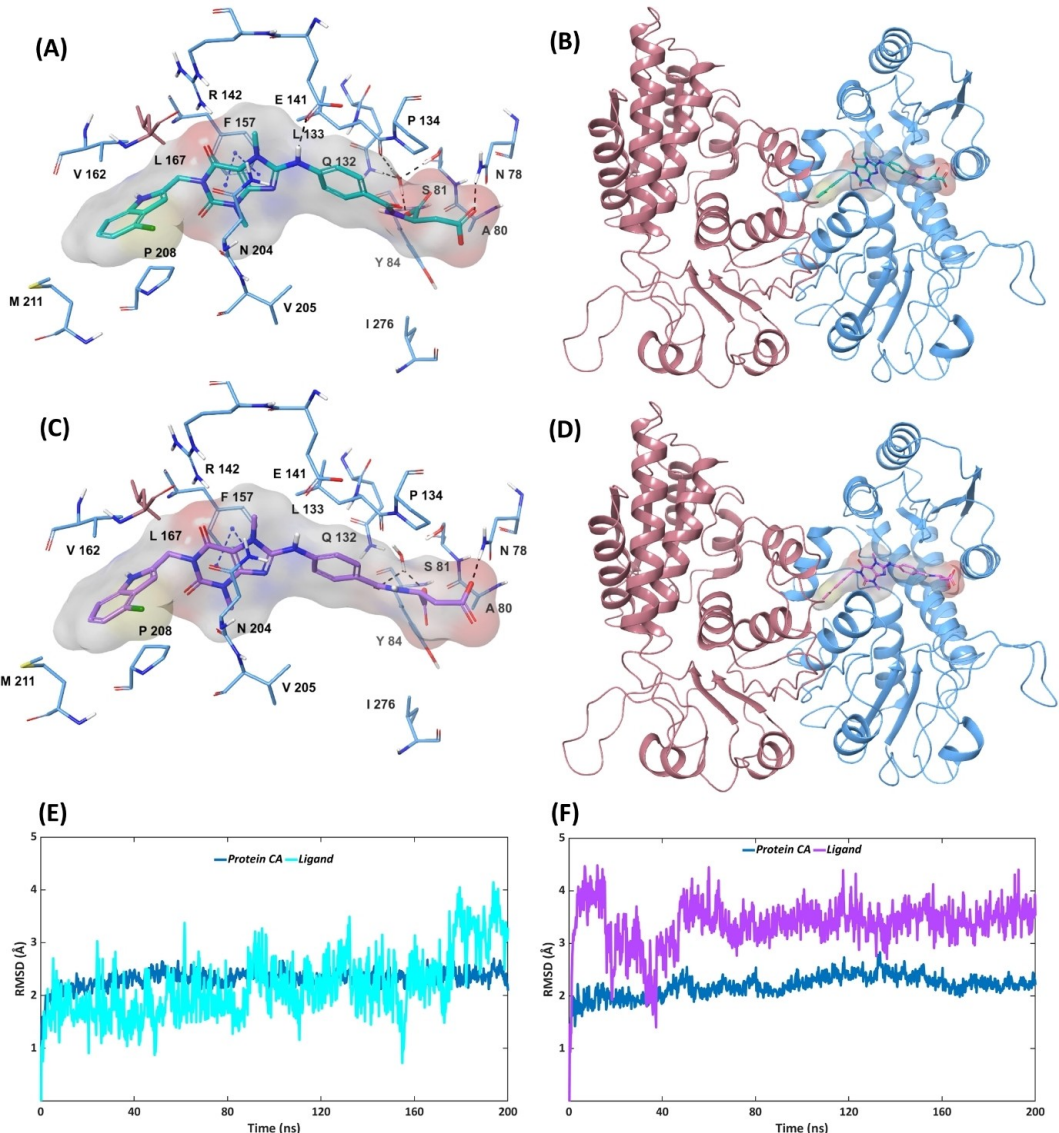
Docking pose (A, B) and RMSD analysis (E) of the MTHFD2‐**4 R** complex. Docking pose (C, D) and RMSD analysis (F) of the MTHFD2‐**4 S** complex.

Experimentally, the best inhibitor among the xanthine‐based derivatives was found to be compound **4 R** with an IC_50_ value of 0.19 μm.^[21]^ Similar to compound **3**, compound **4 R** contains a glutamic acid unit which is supposed to form water‐mediated H‐bond contacts with Ser81, Gln132 and Leu133 of MTHFD2. Since no crystallographic or modeling data was available for compound **4 R**‐MTHFD2 complex, we identified the putative binding pose of not only compound **4 R** but also its enantiomer **4 S** by molecular docking, aimed at investigating whether the *R* enantiomer of compound **4** shows a more promising binding mode and presence of key protein‐ligand interactions over the *S* enantiomer. The reliability of the Glide SP docking program was assessed by performing redocking experiments on the co‐crystallized structures of MTHFD2 (PDB codes: 7EHV, 7EHN and 7EHM). In all cases, the Glide SP docking program was able to reproduce the native crystallographic binding orientation of compounds **1**–**3** with some deviations among the cyclohexanol, propanoic acid and glutamic acid tails of compounds **1**, **2** and **3**, respectively, which seem to be highly flexible and mostly occupy the substrate pocket of MTHFD2 (Figures S1‐S3). As apparent from the suggested docking poses of compounds **4 R** and **4 S** (Figures 6A‐6D), these show similar binding dispositions in MTHFD2 with marginal differences only. Both the enantiomers form π–π stacking with Phe157 as well as water‐mediated H‐bond contacts with Ser81, Gln132 and Leu133. The 4‐chloroindole unit of compounds **4 R**–**4 S** is sandwiched between Val162 and Pro298 of the MTHFD2 allosteric site, similar to compounds **1**–**3**. Due to the incorporation of the 4‐chloroindole unit and the long glutamic acid moiety, the terminal carboxy sidechain of both compound **4 R** and **4 S** is extended towards the solvent‐exposed region and establishes H‐bond interactions with Asn78. Notably, the amino linker of compound **4 R** H‐bonds to the Glu141 sidechain whereas in compound **4 S** this is placed slightly away from Glu141, and thus unable to form this H‐bond interaction.

The MD analysis suggests that compound **4 R** is highly stable during the simulation relative to compound **4 S**, as illustrated in the RMSD plots (Figures 6E, 6F). The lipophilic contacts between compound **4 R** and Phe157, Val162 and Pro208 exist for 100 %, 53 % and 33 % of the simulation time, respectively. A couple of additional lipophilic interactions were also identified, between the phenyl ring of compound **4 R** and Leu133, and between the 4‐chloroindole moiety and Val167 (monomer B), both of which account for 42 % of the simulation trajectory. Furthermore, compound **4 R** was found to facilitate multiple H‐bond interactions with MTHFD2; (a) one of the carbonyl groups from the xanthine core H‐bonds to the Arg142 sidechain for 98 % of the simulation, (b) the nitrogen of the 4‐chloroindole unit H‐bonds to the Asn204 sidechain for 64 % of the simulation time, (c) the Arg201 sidechain forms a H‐bond with the terminal carboxylate sidechain of compound **4 R** during half of the simulation, and also establishes lipophilic contact with the phenyl ring of compound **4 R** for 17 % of the simulation, (d) the terminal carboxylate sidechain of the compound **4 R** glutamic acid forms H‐bond contact with the Arg278 sidechain for 28 % of the simulation, (e) the oxygen backbone of the compound **4 R** glutamic acid H‐bonds to the Tyr84 and Asn78 sidechains for 28 % and 15 % of the simulation time, respectively, and (g) a H‐bond contact between the amino linker of compound **4 R** and the Glu141 sidechain accounts for 17 % of the trajectory (Figures 6A, S11)

Conformational dynamics of compound **4 S** suggest a reduced number/percentage of protein‐ligand contacts in the MTHFD2 allosteric site as well as the presence of a few atypical interactions. Compound **4 S** exerts lipophilic contacts with Phe157, Val162 Pro208 and Leu133 for 96 %, 35 %, 29 % and 28 % of the simulation time, respectively, whereas no van der Waals contacts were noted between the 4‐chloroindole moiety of compound **4 S** and Val167 (monomer B). Interestingly, a new lipophilic contact with Met207 is maintained for 36 % of the simulation. The glutamic acid moiety of compound **4 S** forms lipophilic interaction with the Tyr84 sidechain for 30 % of the simulation, instead of H‐bond contacts, and one of the carbonyl groups from the xanthine core of compound **4 S** (instead of the nitrogen group from 4‐chloroindole) forms H‐bond contact with the Asn204 sidechain, maintained for 66 % of the simulation trajectory. The presence of the H‐bond between the carbonyl on xanthine and the Asn204 sidechain restricts the H‐bond interaction of the same with Arg142 to 22 % of the simulation time. No H‐bond or lipophilic interaction was noted between compound **4 S** and the Arg201 sidechain. Unlike compound **4 R**, the terminal carboxylate sidechain and the backbone oxygen of the compound **4 S** glutamic acid are anchored by Lys88 and Gln132 through H‐bonds contacts for 83 % and 89 % of the simulation time, respectively (Figures 6C, S12). As reported in previous studies,[^19^, ^20^] Lys88 and Gln132 are two important residues providing essential H‐bond contacts to the substrate site binders for MTHFD2 inhibition. The interactions between the glutamic acid unit and the Lys88 and Gln132 residues indicate that compound **4 S** is oriented slightly deeper into the binding pocket, spanning some portions of the MTHFD2 substrate binding site along with the allosteric site. The comparative MD analysis leads to the conclusion that the binding mode and the corresponding protein‐ligand interactions of compound **4 R** are preferred over compound **4 S** for improving the MTHFD2 binding affinity and inhibition (summary of interactions is reported in Table 1). Furthermore, we believe that the gained insights from the stereochemistry of compounds **4 R**–**4 S** could benefit researchers in designing new inhibitors occupying both the substrate and the allosteric site of MTHFD2.

**Table 1:** Protein‐ligand interaction summary of compounds **4 R** and **4 S** from the MD analysis.

Residue	Compound **4** **R**		Compound **4** **S**	
	% Interaction	Type of interaction	% Interaction	Type of Interaction
Asn78	28 %	H‐bond	–	–
Tyr84^[a]^	15 %	H‐bond	30 %	Lipophilic
Lys88^[a]^	–	–	83 %	H‐bond
Gln132^[a]^	–	–	89 %	H‐bond
Leu133	42 %	Lipophilic	28 %	Lipophilic
Glu141	17 %	H‐bond	–	–
Arg142	98 %	H‐bond	22 %	H‐bond
Phe157	100 %	Lipophilic	96 %	Lipophilic
Val162	53 %	Lipophilic	35 %	Lipophilic
Val167^[b]^	42 %	Lipophilic	–	–
Arg201	48 %	H‐bond	–	–
	17 %	Lipophilic	–	–
Asn204	64 %	H‐bond	66 %	H‐bond
Met207	15 %	Lipophilic	36 %	Lipophilic
Pro208	33 %	Lipophilic	29 %	Lipophilic
Arg278	28 %	H‐bond	–	–

[a] Residues belong to the MTHFD2 substrate binding site; [b] residue from monomer B of the MTHFD2 structure (PDB code: 7EHM).

### Verifying the conformational changes induced by the MTHFD2 allosteric inhibitors

**Figure 7:**
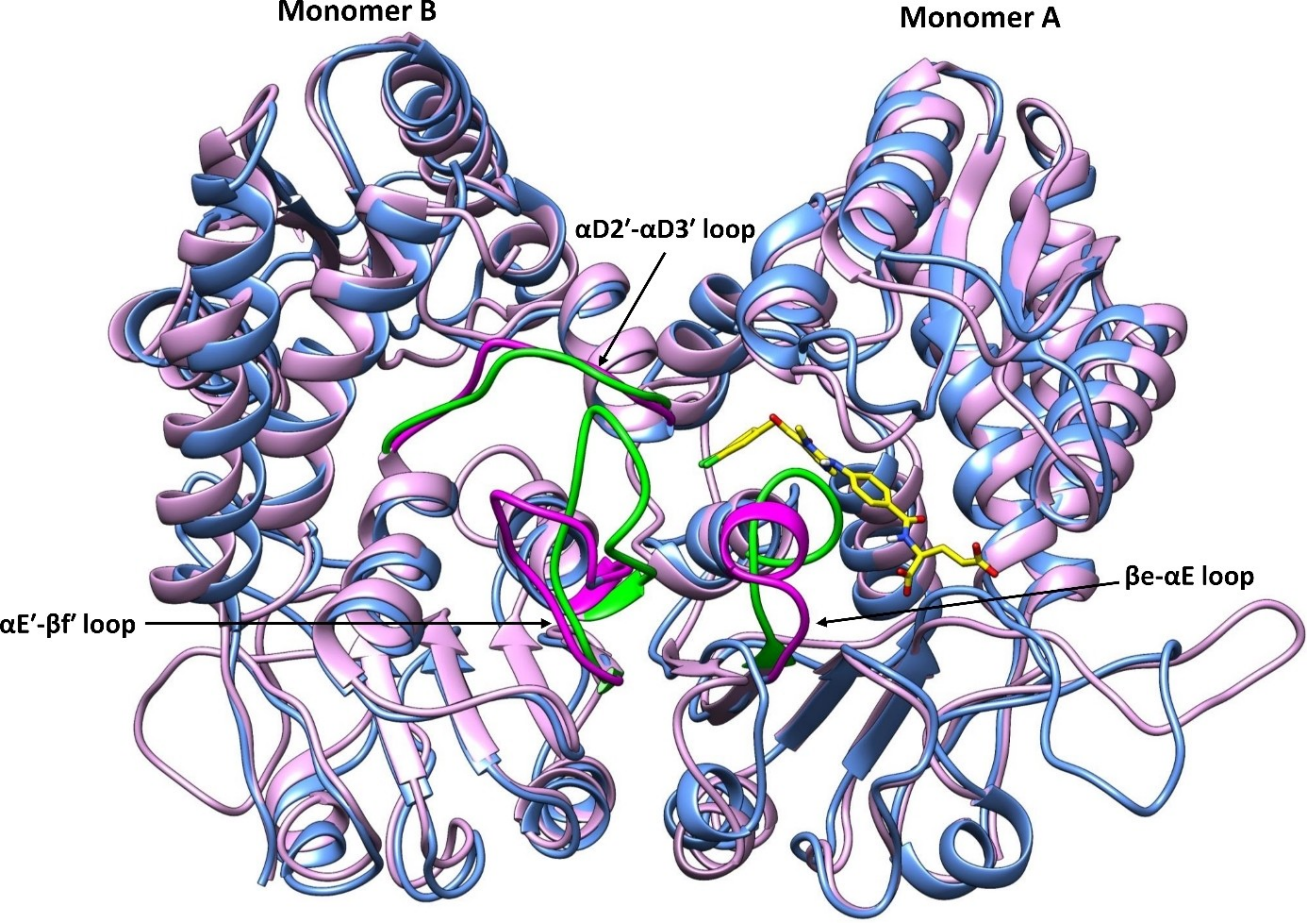
Conformational changes observed from the MD simulations. Superposition of the two representative structures of MTHFD2 from clustering of the MD trajectories are shown. For the MTHFD2‐compound‐**3** complex, the inhibitor is shown in yellow and protein ribbons in blue; the βe‐αE loop, αD2′‐αD3′ loop and αE′‐βf′ loop are colored in magenta. For MTHFD2 without the inhibitor, protein ribbons are in pink and the βe‐αE loop, αD2′‐αD3′ loop and αE′‐βf′ loop are colored in green.

As discussed in the introduction, the co‐crystallized inhibitors (compounds **1**–**3**) induce conformational changes in the βe‐αE loop, the αD2′‐αD3′ loop and the αE′‐βf′ loop of the MTHFD2 allosteric site. We thus analyzed the reported conformational changes in the MD simulation trajectories of the co‐crystallized inhibitors (compounds **1–3**) as well as the docked inhibitors (compounds **4 R**, **4 S**). In addition to the previously mentioned MD analysis of all compounds bound to MTHFD2, we also performed a 200 ns MD simulations on the X‐ray structure of MTHFD2 in absence of any allosteric inhibitor (PDB code: 7EHJ, Figure S18). Five different xanthine‐based inhibitors bound to the allosteric site of MTHFD2 are discussed in this work; however, we have used a representative MD structure of MTHFD2 bound to compound **3**, to compare with a representative MD structure of MTHFD2 when no allosteric inhibitor is bound (Figure 7). The two representative structures were also superposed in order to compare and visualize the displacement of the βe‐αE, αD2′‐αD3′ and αE′‐βf′ loops in the presence of compound **3** (Figure 7). As evident from these figures, only minor displacement occurs of the αD2′‐αD3′ loop over the course of the simulation when the allosteric inhibitor is bound to MTHFD2. In contrast to this, both the βe‐αE loop and the αE′‐βf′ loop were destabilized in presence of compound **3** during the simulation time. The dichlorophenyl head of compound **3** shifts the βe‐αE loop away from the allosteric site of MTHFD2, whereby this undergoes a major conformational change. Similarly, the presence of the dichlorophenyl head and the central xanthine core of compound **3** lead to the displacement of the αE′‐βf′ loop away from the MTHFD2 allosteric site, resulting in a conformational change from loop to helix. The observed conformational changes upon inhibitor binding in the MTHFD2 allosteric site were furthermore analyzed by Root Mean Square Fluctuations (RMSF) analysis. The RMSF of the three loops were calculated for all the MTHFD2‐inhibitor complexes over the course of the simulation, and compared with the one without the allosteric inhibitor The RMSF analysis allowed us to identify which residues among the **3** loops of MTHFD2 that fluctuate the most during the simulation.

The βe‐αE loop which belongs to monomer A of MTHFD2, comprises of 9 amino acid residues; Ala199, Gly200, Arg201, Ser202, Lys203, Asn204, Val205 and Gly206. The αD2′‐αD3′ loop is part of monomer B of MTHFD2 and contains 9 residues; Leu167, Asp168, Gln169, Tyr170, Ser171, Met172, Leu273, Pro174 and Ala175. The αE′‐βf′ loop also belongs to monomer B of MTHFD2, and consists of 14 amino acid residues; His214, Thr215, Asp216, Gly217, Ala218, His219, Glu220, Arg221, Pro222, Gly223, Gly224, Asp225, Ala226 and Thr227. The RMSF of the α‐carbon (Cα), heavy atoms, sidechains and backbones of the above‐mentioned residues from the three loops corresponding to the five MTHFD2‐inhibitor complexes were calculated over the course of the simulation and compared with the structure characterized by the absence of the allosteric inhibitor (Figure S19).

As discussed above, the βe‐αE, αD2′‐αD3′ and αE′‐βf′ loops possess more fluctuation during the simulation when the inhibitors are bound to MTHFD2. As shown in Figure S19, the α‐carbon of Ser202 from the βe‐αE loop was found to be the most fluctuating residue in presence of compound **1** with ∼1.5 Å RMSF, compared to ∼0.2 Å of Ser202 in absence of the allosteric inhibitor. Similarly, the Ser202 backbone of the βe‐αE loop possesses 1.3 Å RMSF in presence of compound **1** compared to the 0.2 Å deviation when no inhibitor binds. Compound **4 S** is another inhibitor causing some notable fluctuations in the βe‐αE loop throughout the simulation. The heavy atoms of Arg201 from the βe‐αE loop shows an RMSF of ∼2.8 Å when compound **4 S** was bound, compared to ∼0.4 Å in absence of any inhibitor. Similarly, the Arg201 sidechain displayed 3.5 Å deviation with compound **4 S** relative to 0.5 Å RMSF without the allosteric inhibitor. Among the α‐carbons of the αD2′‐αD3′ loop, the maximum fluctuation was observed with Ser171 showing ∼1.5 Å RMSF in presence of compound **4 R** relative to ∼0.6 Å RMSF without any inhibitor. The Ser171 backbone of the αD2′‐αD3′ loop shows a deviation of ∼1.5 Å when compound **3** binds to the MTHFD2 allosteric site whereas the RMSF reduces to ∼0.7 Å without the inhibitor.

The heavy atoms of Gln169 in the αD2′‐αD3′ loop show a noteworthy fluctuation of ∼2.5 Å in presence of compound **1**, compared to ∼0.8 Å without the inhibitor. Similarly, the Gln169 sidechain has an RMSF of ∼3.2 Å with compound **1** relative ∼0.9 Å RMSF with no inhibitor. For the αE′‐βf′ loop of MTHFD2, Glu220 was found to be most fluctuated residue showing RMSF values: ∼3.0 Å (Cα), ∼3.7 Å (heavy atoms), ∼4.1 Å (sidechain) and ∼3.6 Å (backbone) over the course of simulation in presence of compound **4 S**, compared to the small deviations: ∼0.3 Å (Cα), ∼1.0 Å (heavy atoms), ∼1.4 Å (sidechain) and ∼1.0 Å (backbone) when the inhibitor is absent. Moreover, His219 is another residue among the αE′‐βf′ loop, which upon binding of compounds **1** and **2** demonstrated a notable increase in the RMSF values relative to when no inhibitor is bound. The α‐carbon of His219 shows RMSF values ∼1.9 Å and ∼2.0 Å during the simulation for compounds **1** and **2**, respectively, but ∼0.3 Å without an inhibitor. Similarly, the His219 heavy atoms fluctuate to ∼2.5 Å and ∼3.0 Å when compounds **1** and **2** are bound to the MTHFD2 allosteric site, respectively, but only ∼0.7 Å in absence of the inhibitor. The sidechain of His219 displays deviation of ∼3.0 Å with compound **1** and ∼3.7 Å with compound **2**, relative to ∼0.8 Å RMSF when no allosteric inhibitor binds. Finally, with respect to the His219 backbone of the αE′‐βf′ loop, RMSF values of ∼1.8 Å and ∼1.7  Å were obtained in the presence of compounds **1** and **2**, respectively, as compared to ∼0.2 Å RMSF in absence of an inhibitor. Taken together, these results from visual inspection of the representative MD structures (Figure 7) and the corresponding RMSF analysis (Figure S19) confirm the reported conformational changes from the crystallographic observations in presence of xanthine‐based compounds at the MTHFD2 allosteric site.

### Docking studies of MTHFD2 inhibitors towards MTHFD1

All the co‐crystallized/published inhibitors (compounds **1**–**3**, **4 R**, **4 S**) discussed in this work are reported as selective binders to the MTHFD2 allosteric site. No crystallographic or modeling data with respect to the MTHFD1 isoform is available for any of these compounds. Thus, we investigated the putative binding mode of all compounds by docking them into the X‐ray structure of MTHFD1 (PDB code: 6ECQ). As opposed to MTHFD2, partial or improper occupancy and poor docking scores are thus anticipated for all compounds binding to MTHFD1 in order to correlate with the experimental data. Figure S4 depicts the suggested docking poses of compounds **1**–**3** in the MTHFD1 allosteric site. Compound **1** adopted a flipped binding orientation and showed absence of any H‐bond interaction with MTHFD1. Leu101 (Leu133 in MTHFD2) was found to be in the vicinity of compound **1**’s xanthine core whereas Ile176 is located close to the oppositely‐oriented cyclohexanol unit of compound **1**. Furthermore, the dichlorophenyl ring system of compound **1** sits next to Tyr52 (Tyr84 in MTHFD2). These three residues (Leu101, Ile276 and Tyr52) could impart lipophilic contacts with compound **1**. One of the chlorine atoms from the dichlorophenyl ring of compound **1** interacts with Thr279 via a halogen bond. The suggested docking pose of compound **2** displays only one H‐bond contact with MTHFD1; the carboxy group of compound **2**’s propanoic acid H‐bonds to Lys56 (Lys88 in MTHFD2), whereas the 4‐chloroindole ring of compound **2** is surrounded by Leu101 (Leu133 in MTHFD2) and Ile176, which could lead to lipophilic contacts. As evident from the putative docking pose, the terminal carboxy sidechain of compound **3**’s glutamic acid moiety H‐bonds to Lys56 (Lys88 in MTHFD2) while its oxygen backbone forms H‐bond interactions with Thr148 and Thr179 in the MTHFD1 allosteric site. The central xanthine core of compound **3** is believed to exhibit lipophilic interactions with Ile176. The docking results of compounds **4 R** and **4 S** suggest the presence of a couple of H‐bond interactions with MTHFD1 (Figure S5). The terminal carboxylate sidechain and the oxygen backbone of the glutamic acid moiety of compound **4 R** are anchored by Lys56 (Lys88 in MTHFD2) and Gln100 (Gln132 in MTHFD2) via H‐bond/salt‐bridge interactions. In the case of compound **4 S**, the terminal carboxy sidechain of the glutamic acid establishes H‐bond/salt‐bridge contacts with Lys56 and Gln100 whereas its oxygen backbone forms H‐bonds with Thr279.

Unlike the MTHFD2 allosteric site, none of the compounds show interactions with the key residues of MTHFD1 that are involved in ligand recognition, that is, Thr111 (Glu141 in MTHFD2), Glu112 (Arg142 in MTHFD2), Leu127 (Phe157 in MTHFD2), Ala132 (Val162 in MTHFD2), Pro180 (Pro208 in MTHFD2). Compounds **1** and **2** show van der Waals contacts with Leu101 in the MTHFD1 allosteric which can be considered mild and marginally contribute to the binding affinity. Furthermore, the lipophilic contact of compound **1** with Tyr52 and H‐bond interactions of compounds **4 R** and **4 S** with Lys56 and Gln100 are notable; however, these residues belong to the substrate binding site of MTHFD1, and thus may not substantially influence the binding at the allosteric site. As apparent from the superposed MTHFD1‐MTHFD2 structures (Figure 3B), the MTHFD1 residues Thr148, Ile176, Thr179 and Thr279 do not seem relevant for the ligand recognition, thus their interactions with some of the compounds are supposed to have marginal impact. Thus, we can conclude that the improper binding poses and the absence of the key protein‐ligand interactions of compounds **1**–**3**, **4 R** and **4 S** contribute to the poor docking scores in MTHFD1 (Table 2), as compared with the desirable binding modes and high docking scores in MTHFD2, justifying their selectivity for the latter.

**Table 2:** Glide scores of all compounds in the MTHFD2 and MTHFD1 binding sites.

Entry	MTHFD2 [−kcal mol^−1^]	MTHFD1 [−kcal mol^−1^]
Compound **1**	−8.68	−5.55
Compound **2**	−13.67	−5.74
Compound **3**	−12.23	−7.14
Compound **4** **R**	−13.10	−7.22
Compound **4** **S**	−12.94	−6.75

### MM‐GBSA binding free energy calculations

In order to validate the desirable binding to MTHFD2 and the poor binding to MTHFD1, all compounds were subjected to binding free energy calculations using the MM‐GBSA method. Along with the binding free energies (Δ*G* Bind), the contributions from Coulomb energy (Δ*G* Coulomb), Lipophilic energy (Δ*G* Lipophilic) and van der Waals energy (Δ*G* van der Waals) of all compounds are enlisted in Table 3 and Table 4 for MTHFD2 and MTHFD1, respectively. Taking the co‐crystallized compound **3** as an example (PDB code: 7EHM), with IC_50_ values of 0.69 μm and >10 μm for MTHFD2 and MTHFD1, respectively, this shows a high binding free energy towards MTHFD2, −82.63 kcal mol^−1^, whereas it displays a significant reduction in Δ*G* Bind (−15.02 kcal mol^−1^) for MTHFD1. Similarly, a marked difference in the Coulomb energy contribution was observed for compound **3** between MTHFD2 (14.05 kcal mol^−1^) and MTHFD1 (111.97 kcal mol^−1^). Furthermore, compound **3** possesses Δ*G* Lipophilic values of −28.20 kcal mol^−1^ and −18.98 kcal mol^−1^ for MTHFD2 and MTHFD1, respectively, again illustrating notable binding free energy difference between the two isoforms. Correspondingly, the van der Waals energy contributions of compound **3** were noted as −79.19 kcal mol^−1^ and −54.66 kcal mol^−1^ for MTHFD2 and MTHFD1, respectively. Taking the energy terms of all compounds together, the MM‐GBSA binding free energy protocol was able to discriminate and validate all the compounds as efficient binders towards MTHFD2 and poor binders to MTHFD1. Despite the notable differences in binding free energies (Δ*G* bind) of all compounds across the two isoforms (MTHFD2 and MTHFD1), the van der Waals energy contributions (Δ*G* van der Waals) of all compounds bound to MTHFD1 only range from −48.36 to −54.66 kcal mol^−1^ (−61.14 to −79.19 kcal mol^−1^ in MTHFD2) and the lipophilic energy contributions (Δ*G* Lipophilic) range between −12.46 to −18.98 kcal mol^−1^ for all compounds in MTHFD1 (−22.34 to −28.20 kcal mol^−1^ in MTHFD2).

**Table 3:** MM‐GBSA binding free energy results (Δ*G* Bind, Δ*G* Coulomb, Δ*G* Lipophilic and Δ*G* van der Waals in kcal mol^−1^) of the compounds co‐crystallized (**1**‐**3**)/docked (**4 R**,**4 S**) to MTHFD2.

Entry	Δ*G* Bind	Δ*G* Coulomb	Δ*G* Lipophilic	Δ*G* van der Waals
Compound **1**	−75.00	−9.17	−27.17	−61.14
Compound **2**	−67.02	−2.95	−22.36	−69.72
Compound **3**	−82.63	14.05	−28.20	−79.19
Compound **4** **R**	−62.15	17.88	−22.34	−74.84
Compound **4** **S**	−55.79	19.87	−22.65	−76.86

**Table 4:** MM‐GBSA binding free energy results (Δ*G* Bind, Δ*G* Coulomb, Δ*G* Lipophilic and Δ*G* van der Waals in kcal mol^−1^) of the compounds docked to MTHFD1.

Entry	Δ*G* Bind	Δ*G* Coulomb	Δ*G* Lipophilic	Δ*G* van der Waals
Compound **1**	−52.37	−6.69	−18.98	−44.47
Compound **2**	−28.57	50.16	−12.46	−43.94
Compound **3**	−15.02	111.97	−18.98	−54.66
Compound **4** **R**	−10.05	114.74	−14.18	−50.68
Compound **4** **S**	−7.67	120.16	−14.88	−48.36

### Induced‐fit docking analysis of all compounds in the MTHFD1 allosteric site

Due to the unavailability of the X‐ray structure of any allosteric inhibitor in complex with MTHFD1 and with the purpose of evaluating the suggested docking poses and MM‐GBSA results, in particular, taking into account the lipophilic and van der Waals energy contributions of all inhibitors in MTHFD1, we also performed Induced‐fit Docking (IFD) of compounds **1**–**3**, **4 R** and **4 S** with respect to the MTHFD1 allosteric site. The IFD protocol enabled us to interrogate the relaxation and conformational changes occurring at the MTHFD1 allosteric site when bound to the MTHFD2 inhibitors. Furthermore, the Glide SP docking poses of all compounds were compared with the IFD poses to evaluate and confirm the undesirable binding in the MTHFD1 allosteric site.

As depicted in the IFD poses (Figure S6) and the IFD results (Table S1), compound **1** shows a flipped binding orientation in the MTHFD1 allosteric site, similar to the Glide docking pose (Figure S4). Compound **1** did not form any interaction with the key residues in the MTHFD1 allosteric. Only Ile176 is located in the proximity of the central xanthine core of compound **1** and the dichlorophenyl unit of compound **1** lies next to Pro102, both of which enable lipophilic contacts. The IFD pose of compound **2** shows the presence of a few interactions with MTHFD1; however, none of them substantially match with the key residues of MTHFD1 allosteric site (Thr111, Glu112, Leu127, Val162 and Pro208). The 4‐chloroindole unit of compound **2** interacts with the Lys175 sidechain via π‐cation contacts. Similar to the IFD pose of compound **1**, the central xanthine core of compound **2** establishes van der Waals contacts with Ile176. The terminal carboxylate from the propanoic acid moiety of compound **2** forms H‐bond/salt‐bridge interaction with Lys56 (Lys88 in MTHFD2). The pyrimidine ring system of compound **2** establishes π–π stacking with the Tyr52 sidechain (Tyr84 in MTHFD2), and one of the nitrogen atoms of the same pyrimidine ring H‐bonds to Gln100 (Gln132 in MTHFD2).

Compound **3** demonstrates multiple interactions with MTHFD1, however the central xanthine core and the phenyl ring of compound **3** are projected away from the key residues in the MTHFD1 allosteric site. The Lys175 sidechain interacts with the dichlorophenyl unit of compound **3** via π‐cation contact and with one of the carbonyl groups from the central xanthine core via H‐bond interaction. One of the chlorine atoms from the dichlorophenyl ring system of compound **3** forms a halogen bond with Asn116 while the other chlorine of the same ring establishes a halogen bond with the Arg137 sidechain. The backbone nitrogen of compound **3**’s glutamic acid unit forms a H‐bond with the Leu101 sidechain (Leu133 in MTHFD2) whereas its backbone oxygen atoms H‐bonds to Gln100 (Gln132 in MTHFD2), Ser49 (Ser81 in MTHFD2) and Arg46. The terminal carboxy sidechain from the glutamic acid of compound **3** is anchored by Lys56 (Lys88 in MTHFD2) and Gln100 (Gln132 in MTHFD2) via H‐bond interactions. None of the enantiomers of compound **4** showed any interactions with the key residues in the allosteric site of MTHFD1 (Figure S7). However, both compounds **4 R** and **4 S** display interactions with some residues, in particular substrate site residues of MTHFD1. One of the carbonyl groups from the central xanthine core of compound **4 R** H‐bonds to the Lys175 sidechain while the chlorine group from the 4‐chlorindole unit of compound **4 R** forms a halogen bond with Arg137 (similar to compound **3**). The backbone oxygen atom from the glutamic acid moiety of compound **4 R** H‐bonds to the sidechains of Lys56 and Gln100, which belong to the substrate site of MTHFD1. In the case of compound **4 S**, the Lys175 sidechain forms π‐cation interactions with the 4‐chloroindole unit instead of the xanthine ring system (as observed with compound **4 R**), which enables the deeper placement of the R‐enantiomer over the S‐enantiomer in the MTHFD1 pocket. The benzoyl carbonyl of compound **4 S** H‐bonds to the Gln100 sidechain (Gln132 in MTHFD2) while the backbone oxygen of the compound **4 S** glutamic acid forms H‐bond/salt‐bridge interactions with the Lys56 sidechain (Lys88 in MTHFD2).

Considering the Glide docking poses and the IFD poses together, all compounds were found to occupy the MTHFD1 allosteric site in a peculiar and partial manner as anticipated. In particular, all inhibitors were oriented away from the key MTHFD1 residues (Leu101, Thr111, Glu112 and Leu127, Ala132 and Pro180), which represent the allosteric site and are involved in ligand recognition. None of the inhibitors display an appropriate binding mode in the MTHFD1 allosteric pocket, nor form any of the corresponding interactions with the key residues. Some of the compounds form interactions with Ser49, Tyr52, Lys56 and Gln100 which are present in the substrate binding site of MTHFD1. Moreover, a few compounds display interactions with Arg46, Asn116, Arg137, Lys175, Ile176 and Ile276 of MTHFD1 which can be considered as trivial and supposed to have only marginal influence on the MTHFD1 inhibition. The gained insights from the desirable poses in MTHFD2 and the poor poses in MTHFD1 can be used to guide the development of selective allosteric inhibitors, taking into consideration the interactions with the key and trivial residues of MTHFD1. In order to explore whether the conformational dynamics of the inhibitors bound to MTHFD1 could eventually lead to desirable binding modes and stable interactions with the key residues at the MTHFD1 allosteric site, we performed MD simulations on the IFD poses of compounds **1**–**3**, **4 R** and **4 S** in complex with MTHFD1.

### MD simulations of MTHFD2 inhibitors bound to the MTHFD1 allosteric site

**Figure 8:**
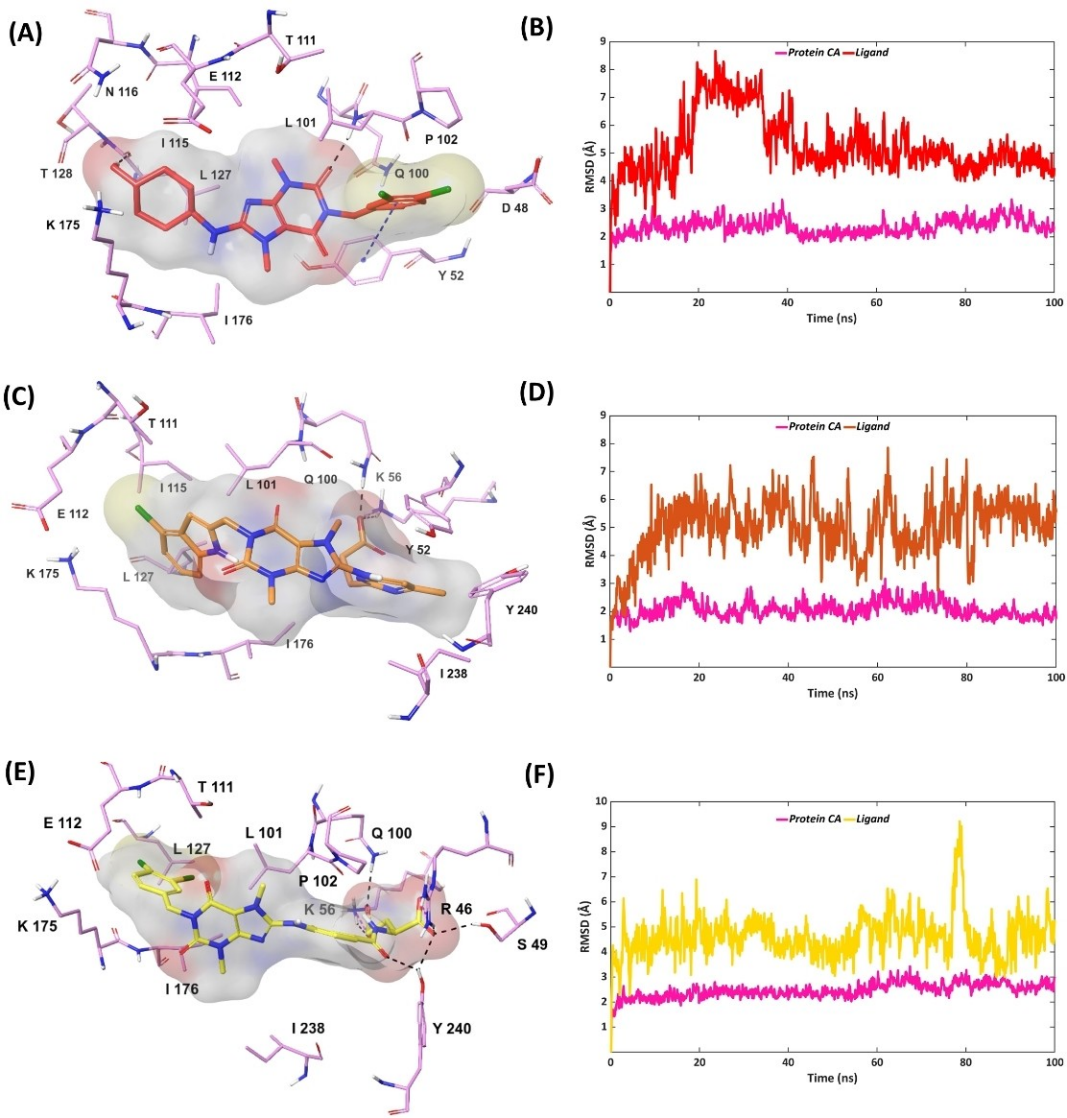
Representative MD structures of MTHFD1‐inhibitor complexes (left column) and RMSD analyses during 100 ns simulations (right column), protein α‐carbons in pink. (A, B) MTHFD1‐compound **1**; (C, D) MTHFD1‐compound **2**; (E, F) MTHFD1‐compound **3**.

The top‐ranked pose generated from the induced‐fit docking of each inhibitor to MTHFD1 was subjected to 100 ns MD simulations. As suggested from both the docking and the IFD poses, compound **1** adopted a flipped binding orientation in the MHTFD1 allosteric site. One of the carbonyl groups from the central xanthine core of compound **1** forms H‐bond interaction with Leu101 which was maintained for 29 % of the simulation time. Furthermore, the xanthine unit of compound **1** interacts with the Leu101 sidechain by lipophilic contacts for 41 % of the simulation. The oppositely‐oriented dichlorophenyl ring of compound **1** forms π–π stacking with Tyr52 which exists during 40 % of the simulation trajectory. The dynamic movement of compound **1** leads to the displacement of the xanthine ring and the cyclohexanol unit further away from the allosteric site, resulting in the formation of a H‐bond interaction between the cyclohexanol hydroxyl of compound **1** and the nitrogen backbone of Leu127, accounting for 68 % of the simulation time (Figures 8A, 8B, S13). No interactions were identified for compound **1** with the other key residues of MTHFD1: Thr111, Gln112, Ala132 and Pro180. The RMSD plot indicates the anticipated ligand movement, representing the movement of the central xanthine ring system of compound **1** away from the MTHFD1 allosteric site, over the course of the simulation. Compound **2** was found to be notably projected towards the substrate binding site of MTHFD1 during the simulation. The 6‐methyl pyrimidine ring of compound **2** establishes π–π stacking with Ty52 for 70 % of the simulation time. The terminal propanoic acid unit of compound **2** interacts with Lys56 for the entire simulation, contributing via H‐bond and salt‐bridge contacts for 64 % and 36 %, respectively. Furthermore, the propanoic acid unit forms a H‐bond with Gln100 throughout the simulation. The interactions with the key residues in the MTHFD1 allosteric site were found to be either negligible or completely absent over the course of simulation, which validate the selectivity of compound **2** for MTHFD2. Compound **2** furthermore shows notable RMSD fluctuation in MTHFD1 during the course of simulation as compared to the MTHFD2 allosteric site (Figures 8C, 8D, S14). The backbone oxygen atom of the terminal glutamic acid unit from compound **3** H‐bonds to Arg46 and Ser49 for 100 % and 63 % of the simulation time, respectively. Of relevance is the fact that Arg46 is not a key residue neither in the allosteric nor the substrate binding site of MTHFD1 whereas Ser49 belongs to the substrate binding site. Furthermore, a new and usual H‐bond interaction between the backbone oxygen of compound **3**‘s glutamic acid unit and Tyr240 was observed for 37 % of the simulation, highlighting the displacement of compound **3** away from the MTHFD1 allosteric site and towards the solvent‐exposed region. The Lys56 sidechain in MTHFD1 interacts with the terminal carboxy sidechain of compound **3** via H‐bond and salt‐bridge interactions which exist for 67 % and 11 % of the simulation time, respectively. Leu101 interacts with compound **3** for 49 % of the simulation via lipophilic and H‐bond contacts. The central xanthine of compound **3** forms lipophilic contacts with the Leu101 sidechain for 38 % of the simulation whereas the backbone oxygen atom of Leu101 H‐bonds with the backbone nitrogen of compound **3**’s glutamic acid moiety for only 11 % of the simulation trajectory. Compound **3** possess increased RMSD values during the simulation time, particularly showing observable movement of its glutamic acid moiety. Apart from the interaction with Leu101, compound **3** did not form interaction with the key residues Thr111, Glu112 and Leu127, Ala132 and Pro180, thus confirming the poor binding in the MTHFD1 allosteric site (Figures 8E, 8F, S15).

**Figure 9:**
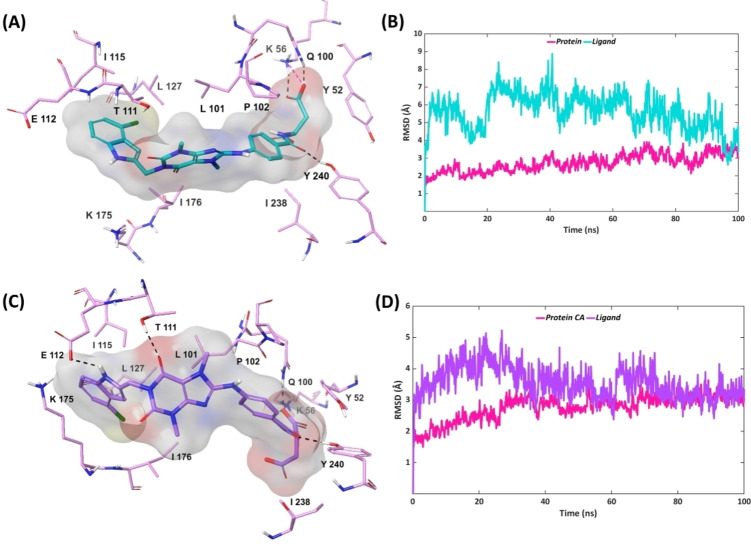
Representative MD structures of MTHFD1‐inhibitor complexes (left column) and RMSD analyses during 100 ns simulations (right column), protein α‐carbons in pink. (A, B) MTHFD1‐compound **4 R**; (C, D) MTHFD1‐compound **4 S**.

On comparing the conformational dynamics of the two enantiomers **4 R** and **4 S**, compound **4 R** possesses higher instability and ligand fluctuation than compound **4 S** over the course of the simulation (Figures 9B, 9D). The terminal carboxy sidechain of compound **4 R's** glutamic acid unit forms H‐bond interaction and salt‐bridge contact with the Lys56 sidechain of MTHFD1 for 88 % and 15 % of the simulation time, respectively (Figures 9A, S16). The said sidechain furthermore H‐bonds to Gln100 which is maintained for 80 % of the simulation trajectory. The long and bulky aliphatic carbon system of compound **4 R's** glutamic acid furthermore establishes lipophilic contact with Tyr52 for 46 % of the simulation. The carbonyl linker connecting the phenyl ring and the glutamic acid unit of compound **4 R** forms H‐bond interactions with Tyr240 for 32 % of the simulation which indicates a movement of the compound towards the solvent‐exposed region, similar to compound **3**. Only interaction with one of the key residues in the MTHFD1 allosteric site was noted for compound **4 R**, a H‐bond contact between one of the carbonyl groups of the xanthine ring and Thr111 for 33 % of the simulation time, while interaction with other key residues were either marginal or missing. For compound **4 S**, the backbone oxygen atom of the glutamic acid, rather than the carboxy sidechain, interacts with Lys56 via H‐bond (83 %) and salt‐bridge (20 %), and with Gln100 for 97 % of the simulation time. The lipophilic interaction between compound **4 S** and Tyr52 is identical to that of compound **4 R**, maintained for 42 % of the trajectory. A new lipophilic interaction between the xanthine core of compound **4 S** and Leu101 was seen for 33 % of the simulation time, which was not observed for compound **4 R**. The carbonyl linker of compound **4 S** H‐bonds to Tyr240 during 88 % of the simulation time (compared to 32 % for compound **4 R**), suggesting a stronger binding of compound **4 S** in the MTHFD1 solvent‐exposed region. Despite the displacement of compound **4 S** away from the allosteric site, the amino group from the 4‐chloroindole unit of compound **4 S** was able to form a H‐bond with Gln112 for 56 % of the simulation, and one of the carbonyl groups from the xanthine core H‐bonds to Thr111 during 59 % of the simulation (Figures 9C, S17). The interactions of compound **4 S** with the two key residues Thr111 and Gln112 validate the stronger binding of compound 4S in MTHFD1 relative to compound **4 R**, further confirming the selectivity towards MTHFD2 of the *R*‐enantiomer as compared to the *S*‐enantiomer.

## Conclusions

In the present work, we have carried out an extensive in silico structural interrogation of the existing xanthine‐based allosteric inhibitors bound to the MTHFD2 protein (co‐crystallized or docked). To the best of our knowledge, the xanthine‐derived compounds are the only reported allosteric inhibitors of MTHFD2 to date. Visual inspection and molecular dynamics (MD) simulation analysis of the co‐crystallized poses of compounds **1**–**3** enabled us to determine desirable binding modes and key protein‐ligand interactions required for MTHFD2 inhibition. The MD simulations of compounds **1**–**3** at the allosteric site of MTHFD2 show significant stability over the course of simulation, which correlates with the experimental binding affinities and corresponding IC_50_ values. Glu112, Arg142, Phe157, Val162 and Pro208 were identified as key residues at the MTHFD2 allosteric site with the inclusion of a few substrate binding site residues (Ser81, Tyr84, Lys88 and Gln132), which altogether contribute to the ligand binding affinity for MTHFD2. Apart from the co‐crystallized inhibitors, compound **4** was reported as the best inhibitor of the series; however, no crystallographic or docking data was available for this compound. Thus, we predicted the binding modes of both enantiomers **4 R** and **4 S** in the MTHFD2 allosteric site, which align well with the co‐crystallized and MD poses of compounds **1**–**3**. The docking and MD analysis revealed that compound **4 R** is preferred over compound **4 S** for the MTHFD2 inhibitory activity, showing desirable binding orientation, key protein‐ligand interactions and relatively stable conformational dynamics. As apparent from the co‐crystallized poses, the xanthine‐based inhibitors reportedly induce conformational changes in the βe‐αE loop (monomer B), αD2′‐αD3′ loop (monomer B) and αE′‐βf′ loop (monomer A) at the MTHFD2 allosteric site, while no conformational changes were observed in absence of the allosteric inhibitor. RMSF analysis of the corresponding MD trajectories of all compounds bound to MTHFD2 were performed and compared with the MTHFD2 X‐ray structure without the allosteric inhibitor, thus validating and confirming the reported conformational changes. Our analysis revealed that the αE′‐βf′ loop showed minor displacement whereas the αD2′‐αD3′ and βe‐αE loops underwent major conformational changes upon binding to the allosteric inhibitors. In particular, Arg201, Ser202 from the βe‐αE loop, Ser171, Gln169 from the αD2′‐αD3′ loop and His219, Glu220 from the αE′‐βf′ loop were found to fluctuate the most during the course of the simulation. Since all compounds were reported as selective inhibitors of MTHFD2 and no crystallographic or docking data was available for any of the compounds binding to MTHFD1, we hypothesized their binding poses on MTHFD1 by docking. The poor docking results of the xanthine‐derived compounds in the MTHFD1 allosteric site were further validated by induced‐fit docking (IFD) which considers receptor flexibility and potential conformational changes. In general, none of the compounds show desirable binding modes or interactions with the key residues of the MTHFD1 allosteric site, neither from docking nor induced‐fit docking poses. Finally, the top‐ranked induced‐fit docking poses of all inhibitors bound to MTHFD1 were inspected by MD simulations which again demonstrated poor binding dispositions, absence of key interactions (with a few exceptions such as compound **4 R** H‐bonding to Thr111) and relatively high ligand instabilities at the MTHFD1 allosteric site compared to the binding to MTHFD2, thus confirming the selective binding and inhibition of MTHFD2 by the xanthine‐based allosteric inhibitors. Our computational protocol which combines docking, IFD and MD simulations seems to be reliable and, therefore, suitable to predict and authenticate the poor binding of the existing/new MTHFD2 inhibitors towards the MTHFD1 isoform to corroborate selectivity. The outcomes of this study are expected to benefit researchers and medicinal chemists working on the rational design of new, potent and selective MTHFD2 inhibitors with anticancer activity.

## Computational Methods

### Protein preparation

The X‐ray crystal structures of MTHFD2 in complex with compound **1** (PDB code: 7EHV), compound **2** (PDB code: 7EHN) or compound **3** (PDB code: 7EHM), or without allosteric inhibitor (PDB code: 7EHJ); and MTHFD1 in complex with LY345899 (PDB code: 6ECQ), were downloaded from the protein data bank.^[23]^ All co‐crystallized structures were prepared using protein preparation wizard,[^24^, ^25^] as implemented in Maestro, Schrödinger.^[26]^ Hydrogen atoms were added and possible metal binding states were generated. The Prime module^[27]^ of Schrödinger was used to add missing atoms, sidechains and loops[^24^, ^28^, ^29^] to the X‐ray complexes, followed by assigning protonation states and generation of tautomeric forms for Asp, Glu, Arg, Lys and His at pH 7.0±2.0. The PROPKA module^[30]^ of Schrödinger was used for H‐bond refinement of all protein structures at pH 7.0. Water molecules with fewer than two hydrogen bonds to non‐waters were removed. In order to fix molecular overlaps and strains, geometry refinements of all protein‐ligand complexes were performed using the OPLS4 force field[^31^, ^32^, ^33^, ^34^] in restrained minimizations until the average root mean square deviation (RMSD) of the protein heavy atoms had converged to 0.3 Å.

### Ligand preparation

The co‐crystallized inhibitors (compounds **1**–**3**) and the reported inhibitor (compound **4** – both *R* and *S* enantiomers) were prepared using the LigPrep module^[35]^ of the Schrödinger suite. Epik[^36^, ^37^, ^38^] (p*K*_a_ prediction tool) was used to assign possible ionization and tautomeric states at pH 7.0±2.0. Energy minimization of the ligands were done using the OPLS4 force field.^[30]^

### Molecular docking

Docking studies were performed with Glide,[^39^, ^40^, ^41^, ^42^] using the ligand structures as prepared above. The prepared protein complex structures of MTHFD2 with compound **3** (PDB code: 7EHM) and MTHFD1 with LY345899 (PDB code: 6ECQ) were used to generate receptor grids for docking into the MTHFD2 and MTHFD1 allosteric sites, respectively. Receptor grids were also generated for the MTHFD2‐compound **1** (PDB code: 7EHV) and MTHFD2‐compound **2** (PDB code: 7EHN) complexes for redocking studies. Cubic receptor grids were generated with regards to the centroid of the bound ligand in the MTHFD2 allosteric site, with a side length of 20 Å. Docking was performed under default settings without receptor grid constraints. The standard precision (SP) mode of Glide was used, which allows for flexible ligand sampling including nitrogen inversions and ring conformations of the ligands. The default parameters of a scaling factor of 0.8 for the van der Waals radii of nonpolar ligand atoms, and partial charge 0.15 cutoff, were used. The docking protocol involves a post‐docking minimization with retention of 30 poses per ligand. The top‐ranked docking poses were selected for each ligand and scored by the default Glide scoring function. The OPLS4 force field^[31]^ was used during the docking process. We performed redocking/self‐docking analysis of the co‐crystallized ligands (compounds **1**–**3**) on their respective MTHFD2 structures (7EHV, 7EHN, 7EHM) to evaluate the reliability of the docking experiment. The Glide SP docking protocol was able to reproduce the crystallographic poses in each case, with marginal RMSD differences.

### Induced‐fit docking

The induced‐fit docking (IFD) program as implemented in Maestro, Schrödinger was used to dock ligands in the MTHFD1 allosteric site. In the IFD protocol, Prime conformational refinement is combined with Glide docking, which initially involves the docking of ligands by the Glide SP mode using a softened potential. The van der Waals scaling factors of both receptor and ligand were set to 0.5, followed by generation of a maximum of 20 poses per ligand. The obtained poses were further subjected to Prime refinement which involves minimization of protein sidechains within 5 Å of the docked ligand, to better accommodate these into the binding site, followed by system minimization using the OPLS4 force field.^[31]^ Finally, the ligands were redocked into the optimized protein structures by Glide SP mode. The IFD poses are ranked based on the following formula [Eq. 1]:

(1) IFDScore=1.0×GlideScore+0.05×PrimeEnergy

The top‐ranked IFD pose of each inhibitor (compounds **1**–**3**, **4 R**, **4 S**) bound to MTHFD1 was selected, visually inspected with respect to the binding mode and protein‐ligand interactions, and further subjected to MD simulations.

### MD simulations and clustering

Classical MD simulations were performed using the Desmond program[^43^, ^44^] in Schrödinger suite 2022–1^[26]^ starting from the crystallographic pose or the putative docking pose of the xanthine‐based inhibitors, with respect to the MTHFD2 or MTHFD1 allosteric sites. A buffer distance of 10 Å was used to solvate the structures with TIP3P^[44]^ water using an orthorhombic box with periodic boundary conditions. In order to adjust the electroneutrality of the system, Na^+^ or Cl^−^ ions were added to give a physiological salt concentration of 0.15 m. The NPT ensemble^[44]^ available within the Desmond package was used for minimization and relaxation of each system under the default settings. The OPLS4 force field^[31]^ was used during all simulations. For the systems involving MTHFD2, each simulation was run for a total of 200 ns with a recording interval of 200 ps, whereas the MD simulations of the top‐ranked induced‐fit docking poses of all inhibitors in MTHFD1 were ran for 100 ns with a recording internal of 100 ps. The temperature and pressure of each system were kept constant at 300 K and 1.01325 bar atmospheric pressure by the Nose‐Hoover thermostat and Martyna‐Tobias‐Klein barostat with isotropic coupling,[^46^, ^47^, ^48^] respectively. Data analysis such as root mean square deviations (RMSD) and protein‐ligand interactions were analyzed using the simulation interaction diagram (SID) panel as implemented in Schrödinger.^[26]^ The obtained trajectories were clustered according to RMSD using the “Desmond Trajectory Clustering” module,^[44]^ setting a frequency value of 10 (every 10^th^ ns) and up to a maximum of 10 clusters. The most populated clusters were used as representative structure for the MTHFD2 or MTHFD1 inhibitor complexes.

### MM‐GBSA binding free energy calculation

Molecular mechanics with generalized Born and surface area solvation (MM‐GBSA) is a largely used physics‐based method to estimate the binding free energy of a ligand bound to a protein.^[49]^ The Prime module^[27]^ in the Schrödinger suite 2022‐1^[26]^ was used to compute MM‐GBSA free energy of binding (Δ*G* bind) for all compounds bound to MTHFD2 or MTHFD1, using the following equation [Eq. (2)]: 2

(2) $$\Delta G\;bind=E{}_{Complex}-E{}_{Ligand}-E{}_{Receptor}$$

where *E_Complex_*, *E_Ligand_*, and *E_Receptor_* represent the energy calculations carried out in the Prime MM GBSA module for the optimized complex (complex), optimized free ligand (ligand), and optimized free receptor (receptor), respectively. The OPLS4 force field^[30]^ and VSGB solvation model^[50]^ were employed in the binding free energy calculations. The obtained binding free energies (Δ*G* Bind), the Coulomb energy contribution (Δ*G* Coulomb), the Lipophilic energy (Δ*G* Lipophilic) and the van der Waals energy (Δ*G* van der Waals) of all compounds are discussed in this work.

## Supporting Information

The enclosed Supporting Information contains crystallographic binding poses and redocking poses of compounds **1**–**3** in MTHFD2, docking poses and induced fit docking poses of all inhibitors in MTHFD1, protein‐ligand interaction histograms from the MD simulations of all compounds with respect to MTHFD2 and MTHFD1, and induced‐fit docking results of all inhibitors in MTHFD1.

## Author contributions

V. J. and L. A. E. formulated the study. V. J. performed the computations, analyzed the data and wrote the initial version of the manuscript. L. A. E. revised the text as final version of the manuscript.

## Conflict of interest

The authors declare no competing interests.

1

## Supporting information

As a service to our authors and readers, this journal provides supporting information supplied by the authors. Such materials are peer reviewed and may be re‐organized for online delivery, but are not copy‐edited or typeset. Technical support issues arising from supporting information (other than missing files) should be addressed to the authors.

Supporting Information

## Data Availability

The structures of the docked complexes, induced‐fit docking complexes and the MD trajectory files of compounds **1**–**3**, **4 R** and **4 S** with respect to MTHFD2 and MTHFD1, are provided as tarballs (.tar.gz) freely accessible at https://zenodo.org/, through DOI: 10.5281/zenodo.7454250.
