# Supplementary material for: Binding Modes of Xanthine‐Derived Selective Allosteric Site Inhibitors of MTHFD2
Source: ChemistryOpen. 2023 May 2;12(5):e202300052. doi: 10.1002/open.202300052 (PMC10152887; doi:10.1002/open.202300052)
Supplement: Supplementary file 1 — Supporting Information [file OPEN-12-e202300052-s001.pdf]

ChemistryOpen

Supporting Information

Binding Modes of Xanthine-Derived Selective Allosteric Site Inhibitors of MTHFD2

Vibhu Jha and Leif A. Eriksson*

Table of contents

Figure S1. Redocking pose of compound 1 in the MTHFD2 allosteric site, compared with the co-crystallized pose. P3

Figure S2. Redocking pose of compound 2 in the MTHFD2 allosteric site, compared with the co-crystallized pose. P3

Figure S3. Redocking pose of compound 3 in the MTHFD2 allosteric site, compared with the co-crystallized pose. P4

Figure S4. Docking poses of compounds 1-3 in the MTHFD1 allosteric site. P4

Figure S5. Docking poses of compounds 4R-4S in the MTHFD1 allosteric site P5

Figure S6. Induced-fit docking poses of compounds 1-3 in the MTHFD1 allosteric site. P5

Figure S7. Induced-fit docking poses of compounds 4R-4S in the MTHFD1 allosteric site. P6

Figure S8. Protein-ligand interaction histogram from the MD simulations of compound 1 in the MTHFD2 allosteric site. P6

Figure S9. Protein-ligand interaction histogram from the MD simulations of compound 2 in the MTHFD2 allosteric site. P7

Figure S10. Protein-ligand interaction histogram from the MD simulations of compound 3 in the MTHFD2 allosteric site. P7

Figure S11. Protein-ligand interaction histogram from the MD simulations of compound 4R in the MTHFD2 allosteric site. P8

Figure S12. Protein-ligand interaction histogram from the MD simulations of compound 4S in the MTHFD2 allosteric site. P8

Figure S13. Protein-ligand interaction histogram from the MD simulations of compound 1 in the MTHFD1 allosteric site. P9

Figure S14. Protein-ligand interaction histogram from the MD simulations of compound 2 in the MTHFD1 allosteric site. P9

Figure S15. Protein-ligand interaction histogram from the MD simulations of compound 3 in the MTHFD1 allosteric site. P10

Figure S16. Protein-ligand interaction histogram from the MD simulations of compound 4R in the MTHFD1 allosteric site. P10

Figure S17. Protein-ligand interaction histogram from the MD simulations of compound 4S in the MTHFD1 allosteric site. P11

Figure S18. RMSD analysis from the 200 ns MD simulations of MTHFD2 X-ray structure without the allosteric inhibitor. P11

Figure S19. RMSF analysis of the conformational changes at the MTHFD2 allosteric site in the presence of compounds 1-3, 4R and 4S, versus structures in the absence of inhibitor, as observed from the MD simulations. P12-13

Figure S20. Crystallographic binding mode of the folate-based inhibitor LY345899 in the substrate binding site of MTHFD1. P13

Table S1. Induced-fit docking results of all compounds in the MTHFD1 allosteric site. P14

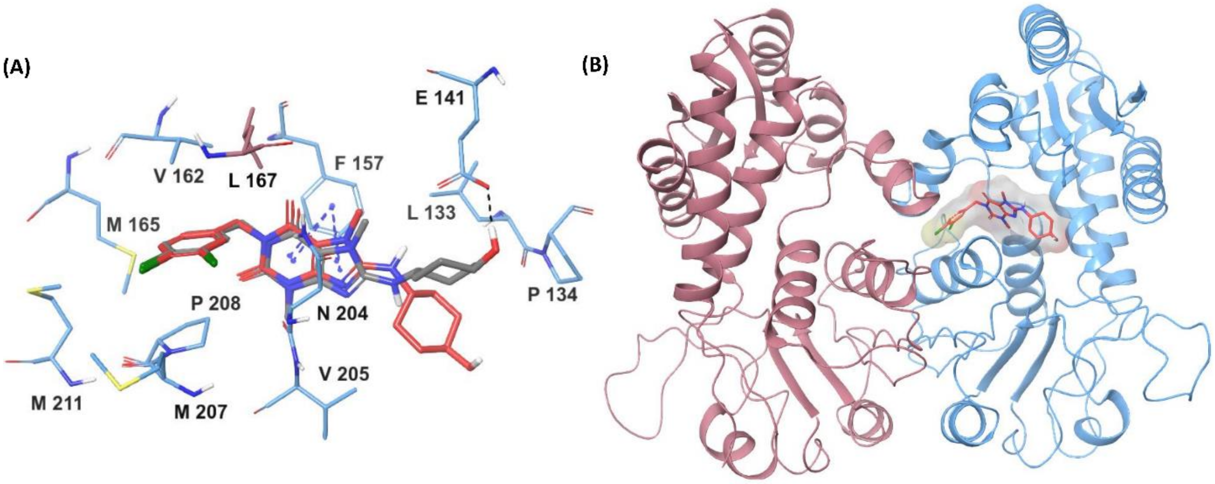

Figure S1. (A) Redocking pose of compound 1 in the MTHFD2 allosteric site (docked pose in grey, co-crystallized pose in red, protein residues: blue – monomer A, brown – monomer B, PDB code: 7EHV). **(B)** Ribbon view: co-crystallized pose of compound 1 (red) in the MTHFD2 allosteric site (blue ribbons – monomer A, brown ribbons – monomer B).

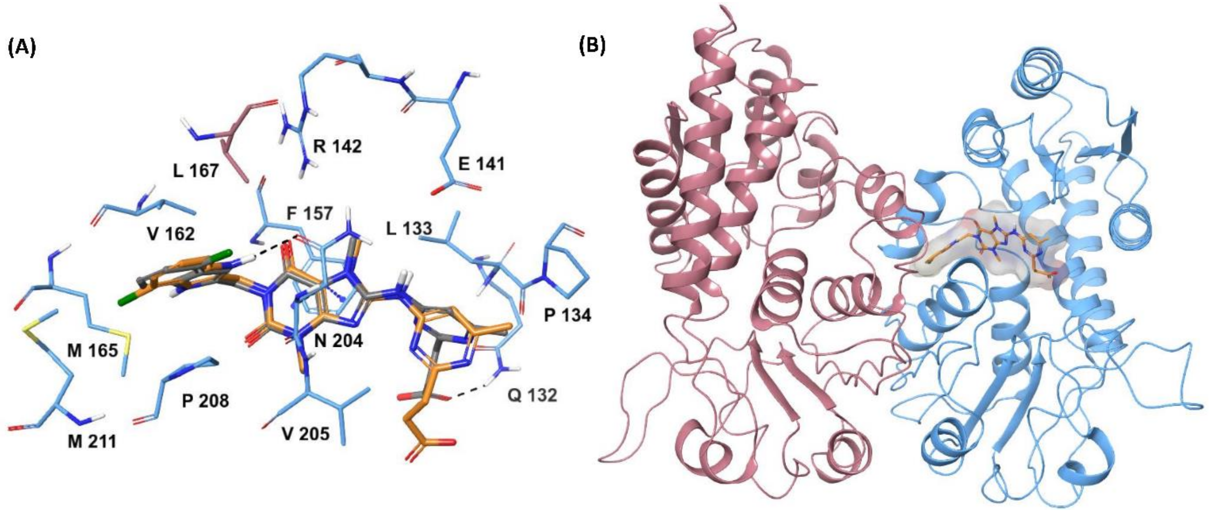

Figure S2. (A) Redocking pose of compound 2 in the MTHFD2 allosteric site (docked pose in grey, co-crystallized pose in orange, protein residues: blue – monomer A, brown – monomer B, PDB code: 7EHN). **(B)** Ribbon view: co-crystallized pose of compound 2 (orange) in the MTHFD2 allosteric site (blue ribbons – monomer A, brown ribbons – monomer B).

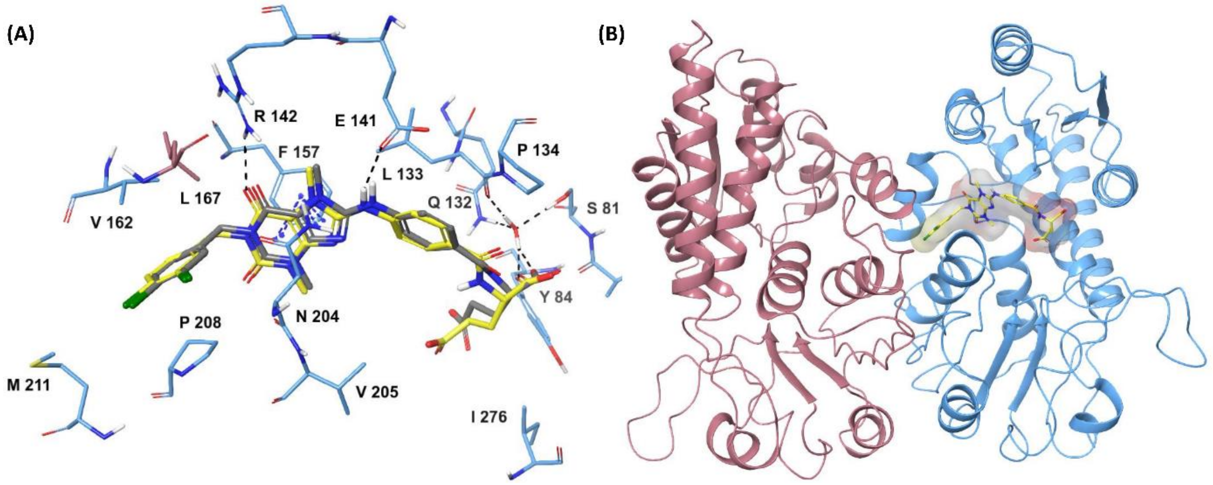

Figure S3. (A) Redocking pose of compound 3 in the MTHFD2 allosteric site (docked pose in grey, co-crystallized pose in yellow, protein residues: blue – monomer A, brown – monomer B, PDB code: 7EHM). **(B)** Ribbon view: co-crystallized pose of compound 3 (orange) in the MTHFD2 allosteric site (blue ribbons – monomer A, brown ribbons – monomer B).

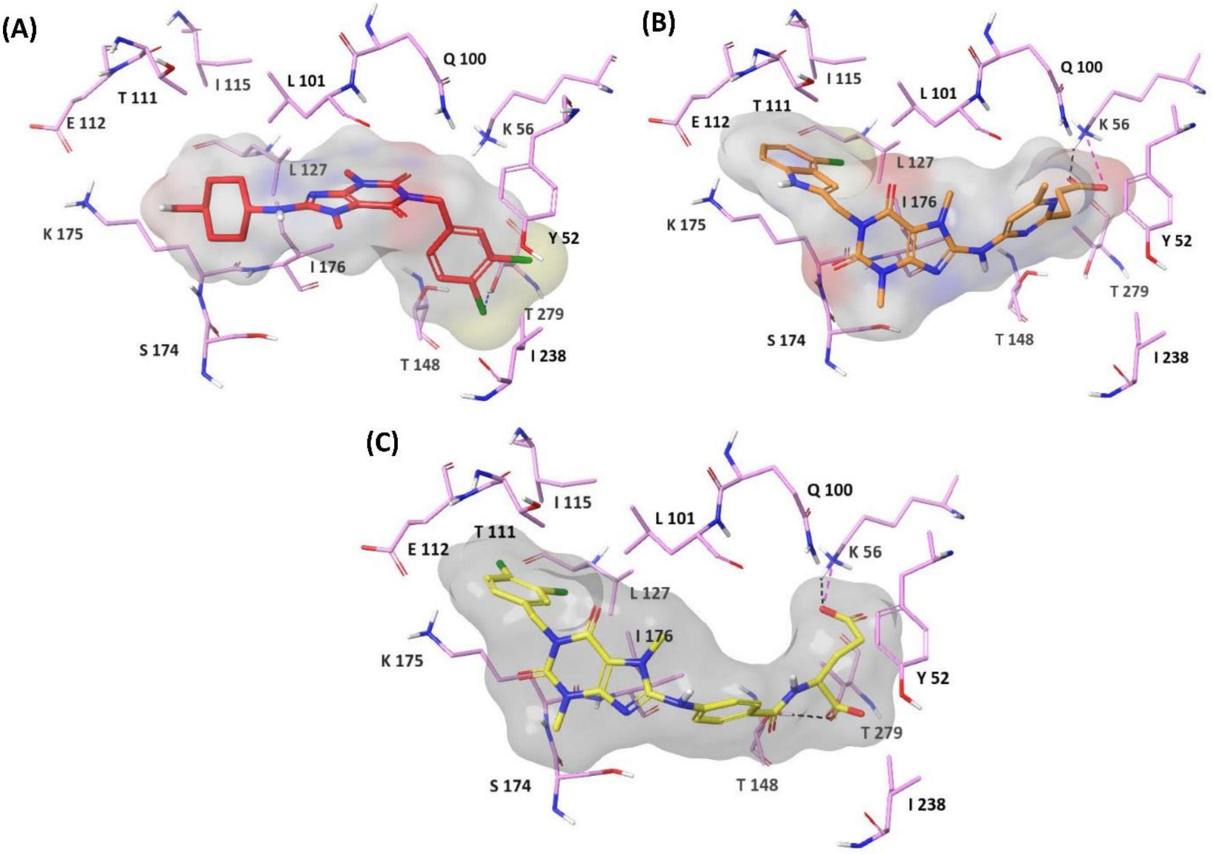

Figure S4. Docking poses of compound 1, 2 and 3 in the MTHFD1 allosteric site **(A)** Compound 1 in red. **(B)** Compound 2 in orange. **(C)** Compound 3 in yellow. MTHFD1 protein residues are colored in pink (PDB code: 6ECQ).

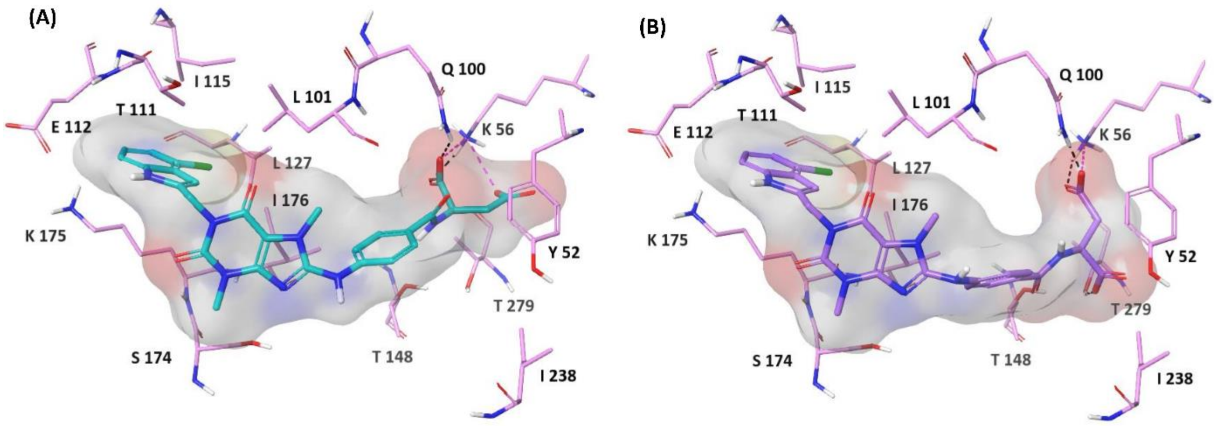

Figure S5. Docking poses of compound 4R and 4S in the MTHFD1 allosteric site **(A)** Compound 4R in cyan. **(B)** Compound 4S in purple. MTHFD1 protein residues are colored in pink (PDB code: 6ECQ).

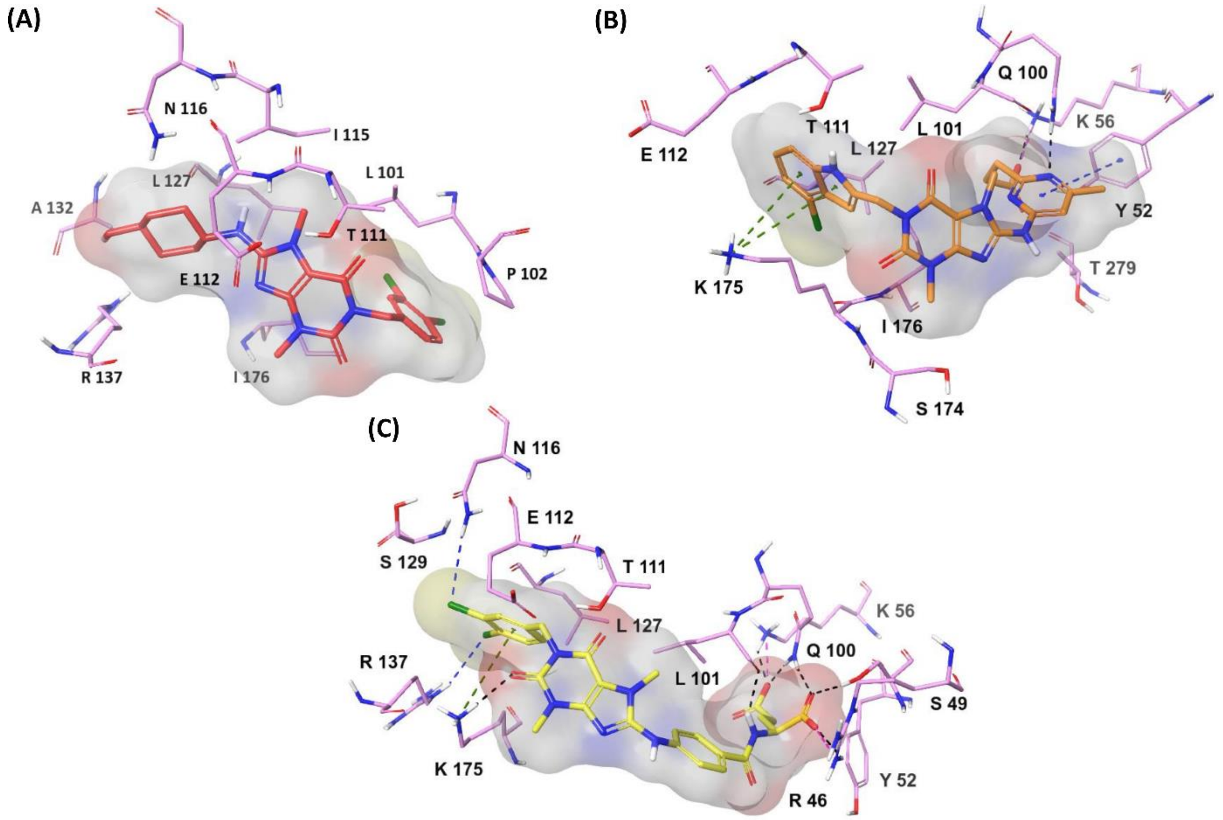

Figure S6. Induced-fit docking poses of compound 1, 2 and 3 in the MTHFD1 allosteric site **(A)** Compound 1 in red. **(B)** Compound 2 in orange. **(C)** Compound 3 in yellow. MTHFD1 protein residues are colored in pink (PDB code: 6ECQ).

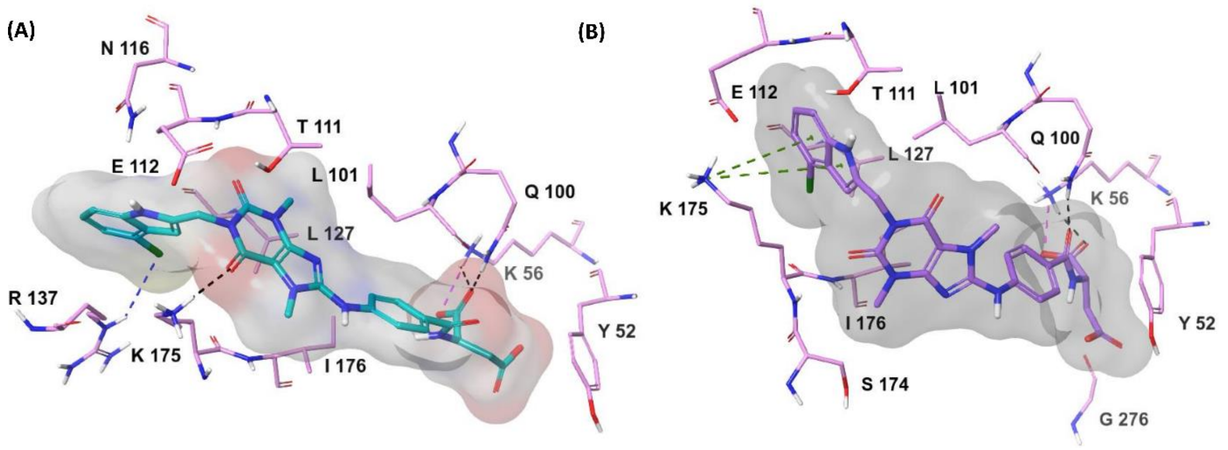

Figure S7. Induced-fit docking poses of compound 4R and 4S in the MTHFD1 allosteric site **(A)** Compound 4R in cyan. **(B)** Compound 4S in purple. MTHFD1 protein residues are colored in pink (PDB code: 6ECQ).

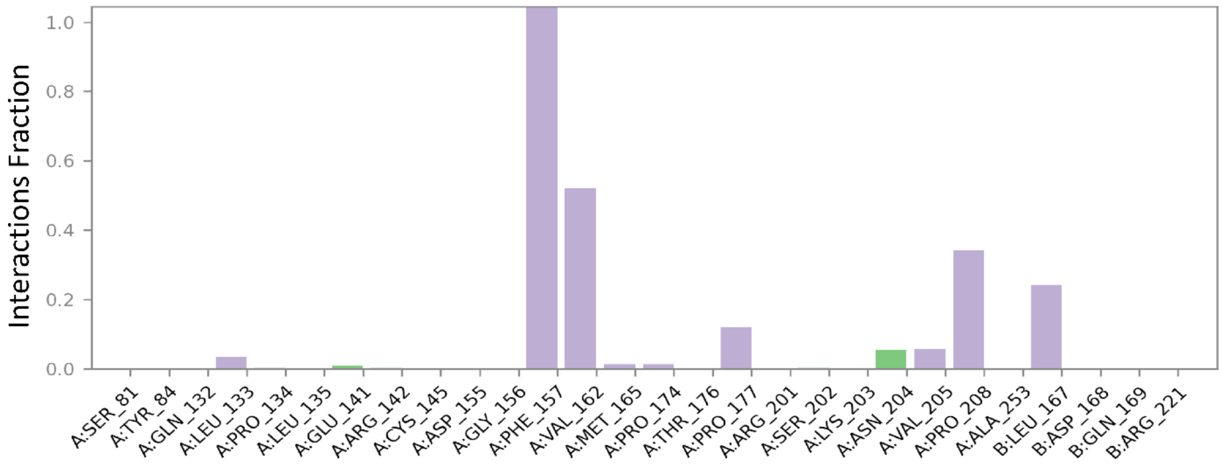

Figure S8. Protein-ligand interaction histogram from the MD simulations of compound 1 in the MTHFD2 allosteric site. (H-bonds are shown in green, salt-bridge interactions are shown in pink, and lipophilic contacts are shown in grey).

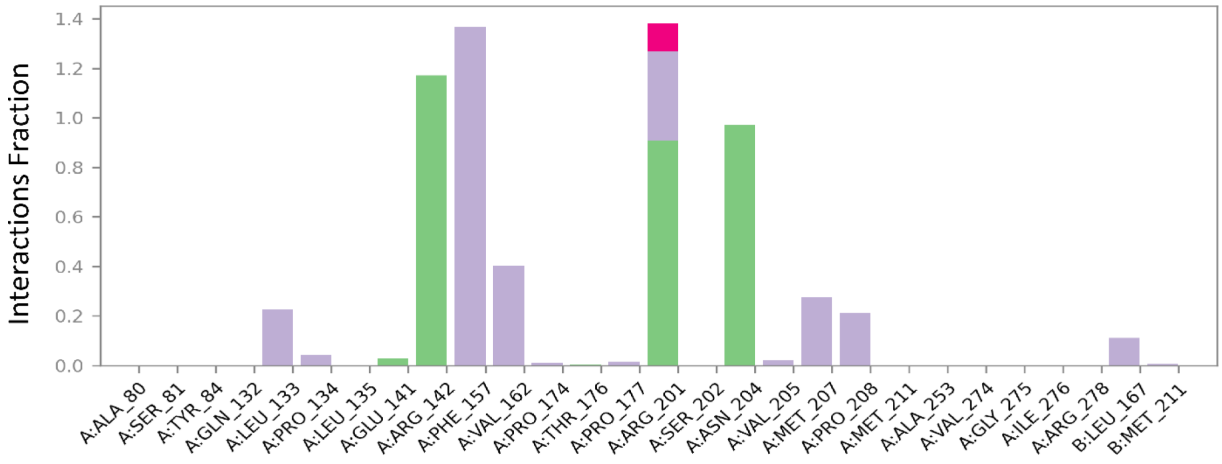

Figure S9. Protein-ligand interaction histogram from the MD simulations of compound 2 in the MTHFD2 allosteric site. (H-bonds are shown in green, salt-bridge interactions are shown in pink, and lipophilic contacts are shown in grey).

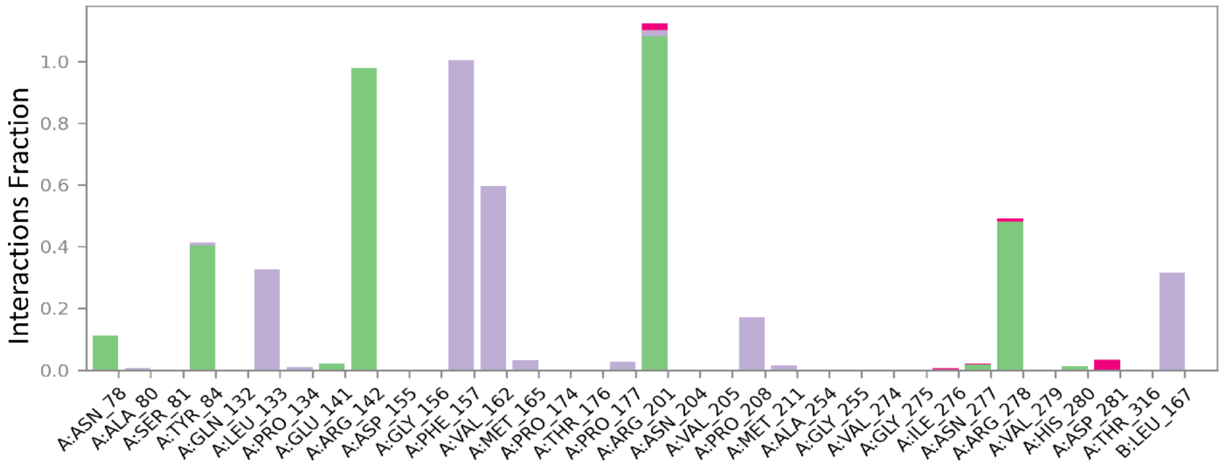

Figure S10. Protein-ligand interaction histogram from the MD simulations of compound 3 in the MTHFD2 allosteric site. (H-bonds are shown in green, salt-bridge interactions are shown in pink, and lipophilic contacts are shown in grey).

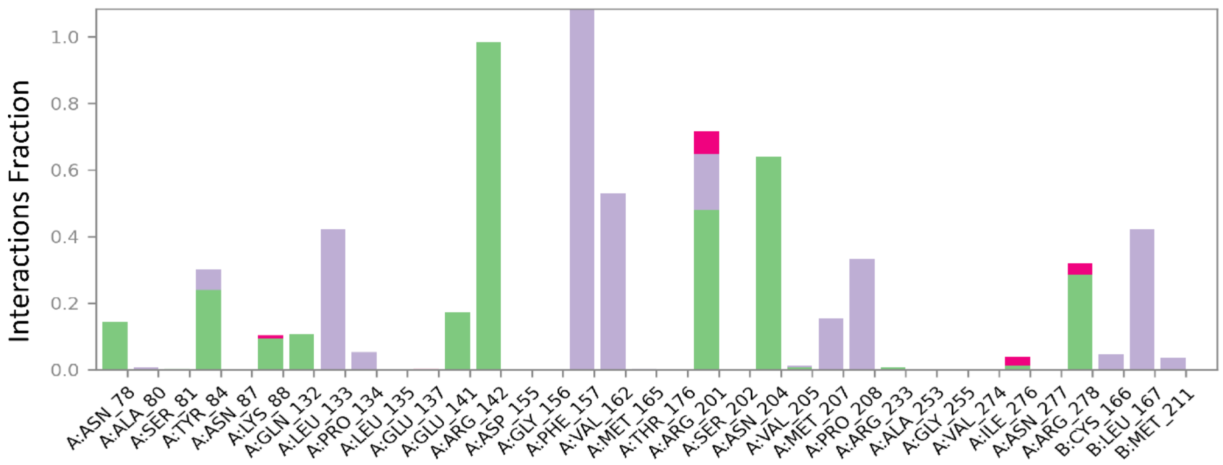

Figure S11. Protein-ligand interaction histogram from the MD simulations of compound 4R in the MTHFD2 allosteric site. (H-bonds are shown in green, salt-bridge interactions are shown in pink, and lipophilic contacts are shown in grey).

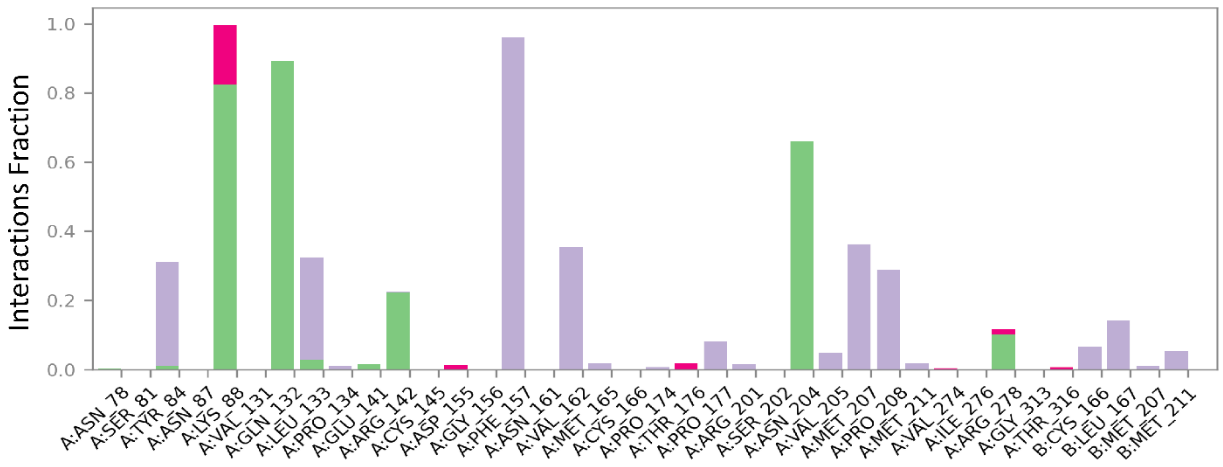

Figure S12. Protein-ligand interaction histogram from the MD simulations of compound 4S in the MTHFD2 allosteric site. (H-bonds are shown in green, salt-bridge interactions are shown in pink, and lipophilic contacts are shown in grey).

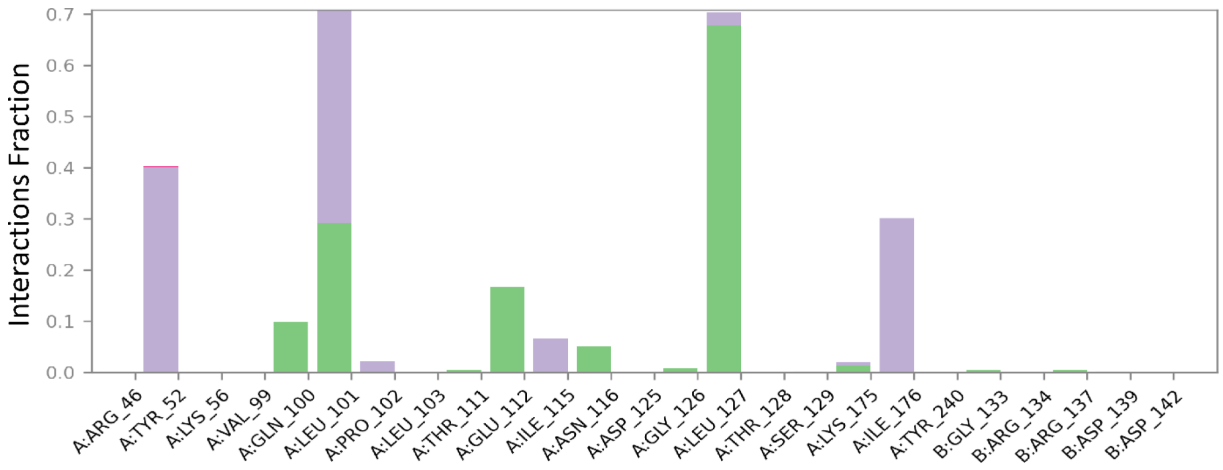

Figure S13. Protein-ligand interaction histogram from the MD simulations of compound 1 in the MTHFD1 allosteric site. (H-bonds are shown in green, salt-bridge interactions are shown in pink, and lipophilic contacts are shown in grey).

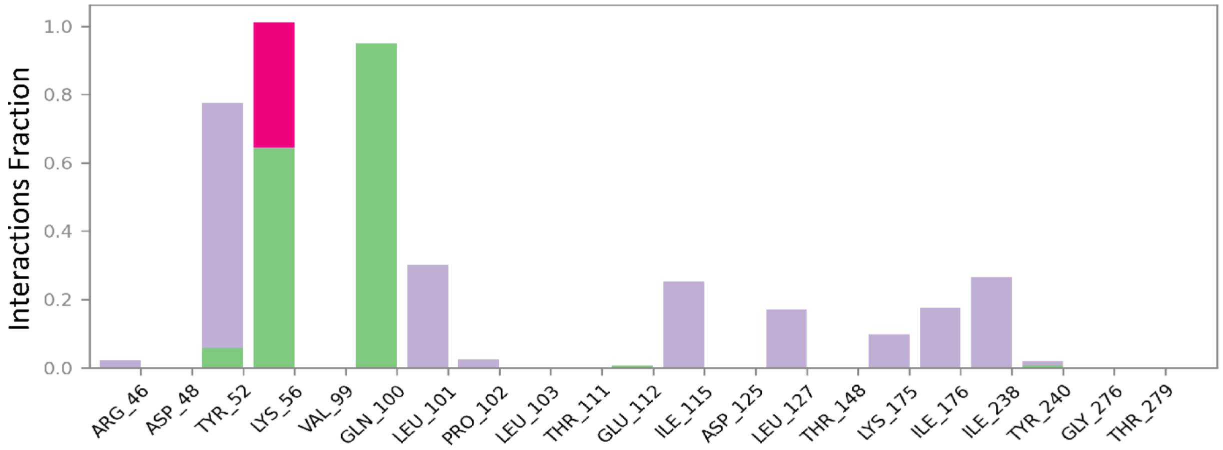

Figure S14. Protein-ligand interaction histogram from the MD simulations of compound 2 in the MTHFD1 allosteric site. (H-bonds are shown in green, salt-bridge interactions are shown in pink, and lipophilic contacts are shown in grey).

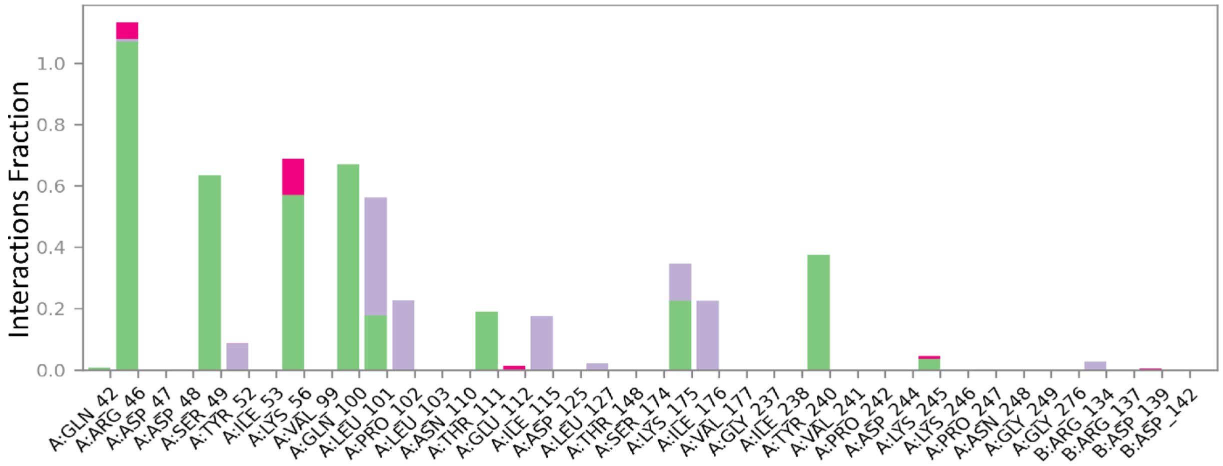

Figure S15. Protein-ligand interaction histogram from the MD simulations of compound 3 in the MTHFD1 allosteric site. (H-bonds are shown in green, salt-bridge interactions are shown in pink, and lipophilic contacts are shown in grey).

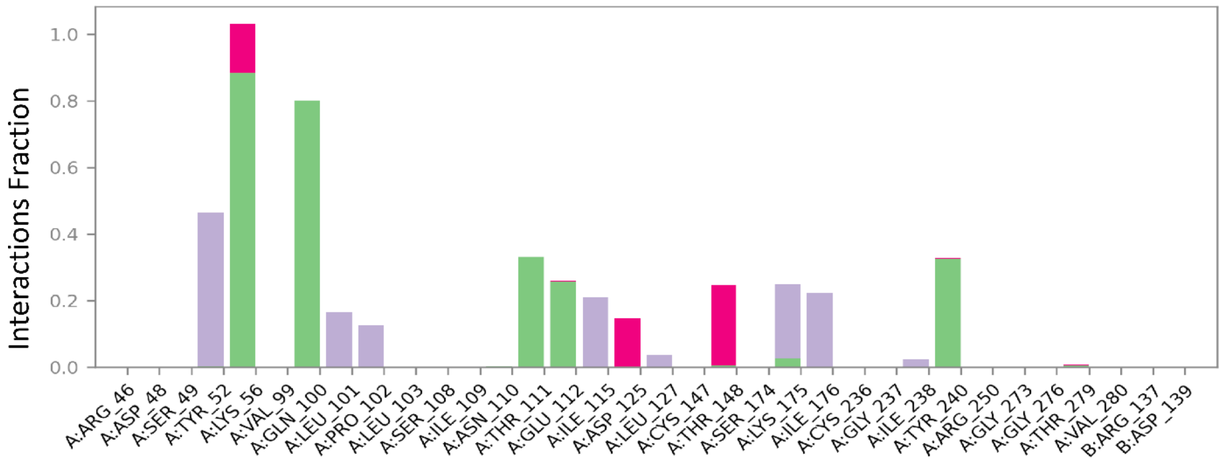

Figure S16. Protein-ligand interaction histogram from the MD simulations of compound 4R in the MTHFD1 allosteric site. (H-bonds are shown in green, salt-bridge interactions are shown in pink, and lipophilic contacts are shown in grey).

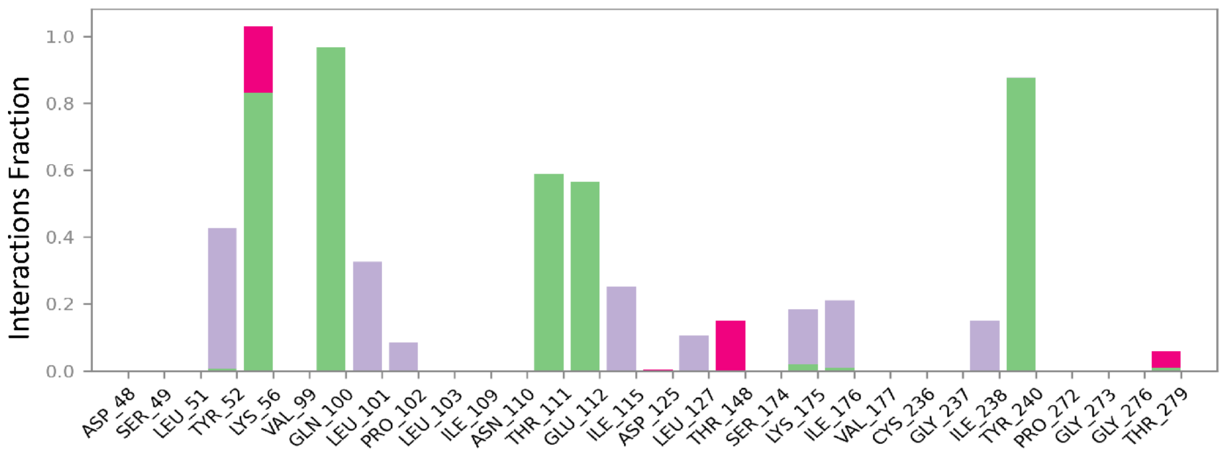

Figure S17. Protein-ligand interaction histogram from the MD simulations of compound 4S in the MTHFD1 allosteric site. (H-bonds are shown in green, salt-bridge interactions are shown in pink, and lipophilic contacts are shown in grey).

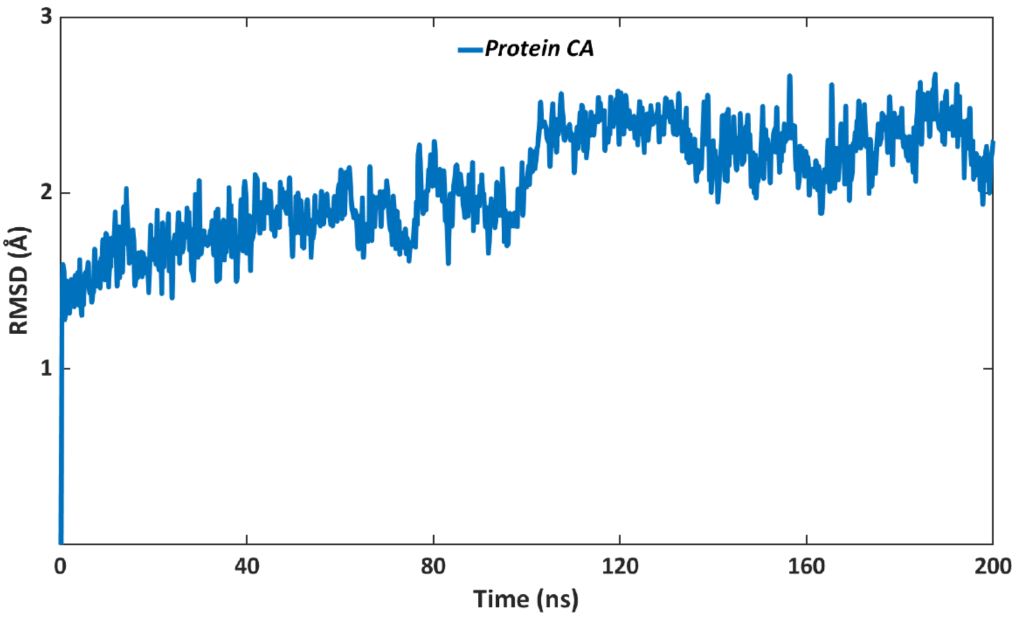

Figure S18. RMSD analysis from the 200 ns MD simulations of MTHFD2 X-ray structure (PDB code: 7EHJ) without the allosteric inhibitor (protein α-carbons).

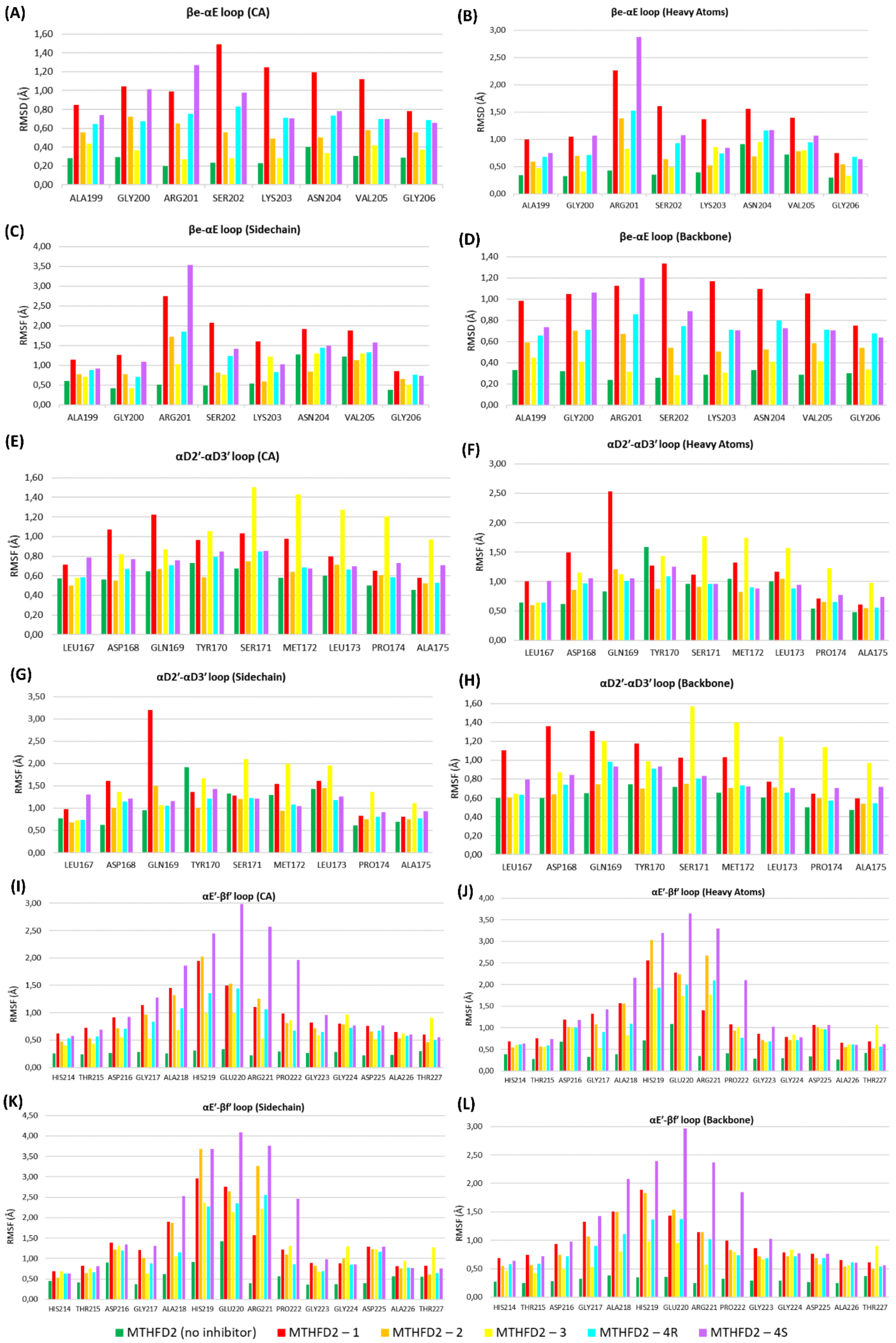
(A)
βe-αE loop (CA)
RMSD (Å)
ALA199 GLY200 ARG201 SER202 LYS203 ASN204 VAL205 GLY206
(B)
βe-αE loop (Heavy Atoms)
RMSD (Å)
(C)
βe-αE loop (Sidechain)
RMSF (Å)
(D)
βe-αE loop (Backbone)
RMSD (Å)
(E)
αD2′-αD3′ loop (CA)
RMSF (Å)
LEU167 ASP168 GLN169 TYR170 SER171 MET172 LEU173 PRO174 ALA175
(F)
αD2′-αD3′ loop (Heavy Atoms)
RMSF (Å)
(G)
αD2′-αD3′ loop (Sidechain)
RMSF (Å)
(H)
αD2′-αD3′ loop (Backbone)
RMSF (Å)
(I)
αE′-βf′ loop (CA)
RMSF (Å)
HIS214 THR215 ASP216 GLY217 ALA218 HIS219 GLU220 ARG221 PRO222 GLY223 GLY224 ASP225 ALA226 THR227
(J)
αE′-βf′ loop (Heavy Atoms)
RMSF (Å)
(K)
αE′-βf′ loop (Sidechain)
RMSF (Å)
(L)
αE′-βf′ loop (Backbone)
RMSF (Å)
MTHFD2 (no inhibitor) MTHFD2 – 1 MTHFD2 – 2 MTHFD2 – 3 MTHFD2 – 4R MTHFD2 – 4S

Figure S19. RMSF analysis of the conformational changes at the MTHFD2 allosteric site in the presence of compounds 1-3, 4R and 4S, versus structures in the absence of inhibitor, as observed from the MD simulations. (A) βe-αE loop (Cα). (B) βe-αE loop (Heavy Atoms). (C) βe-αE loop (Sidechain). (D) βe-αE loop (Backbone). (E) αD2′-αD3′ loop (Cα). (F) αD2′-αD3′ loop (Heavy atoms). (G) αD2′-αD3′ loop. (Sidechain). (H) αD2′-αD3′ loop (Backbone). (I) αE′-βf′ loop (Cα). (J) αE′-βf′ loop (Heavy Atoms). (K) αE′-βf′ loop (Sidechain). (L) αE′-βf′ loop (Backbone). MTHFD2 structure without inhibitor is colored in green. MTHFD2 structures with compounds 1, 2, 3, 4R and 4S are colored in red, orange, yellow, cyan and purple, respectively.

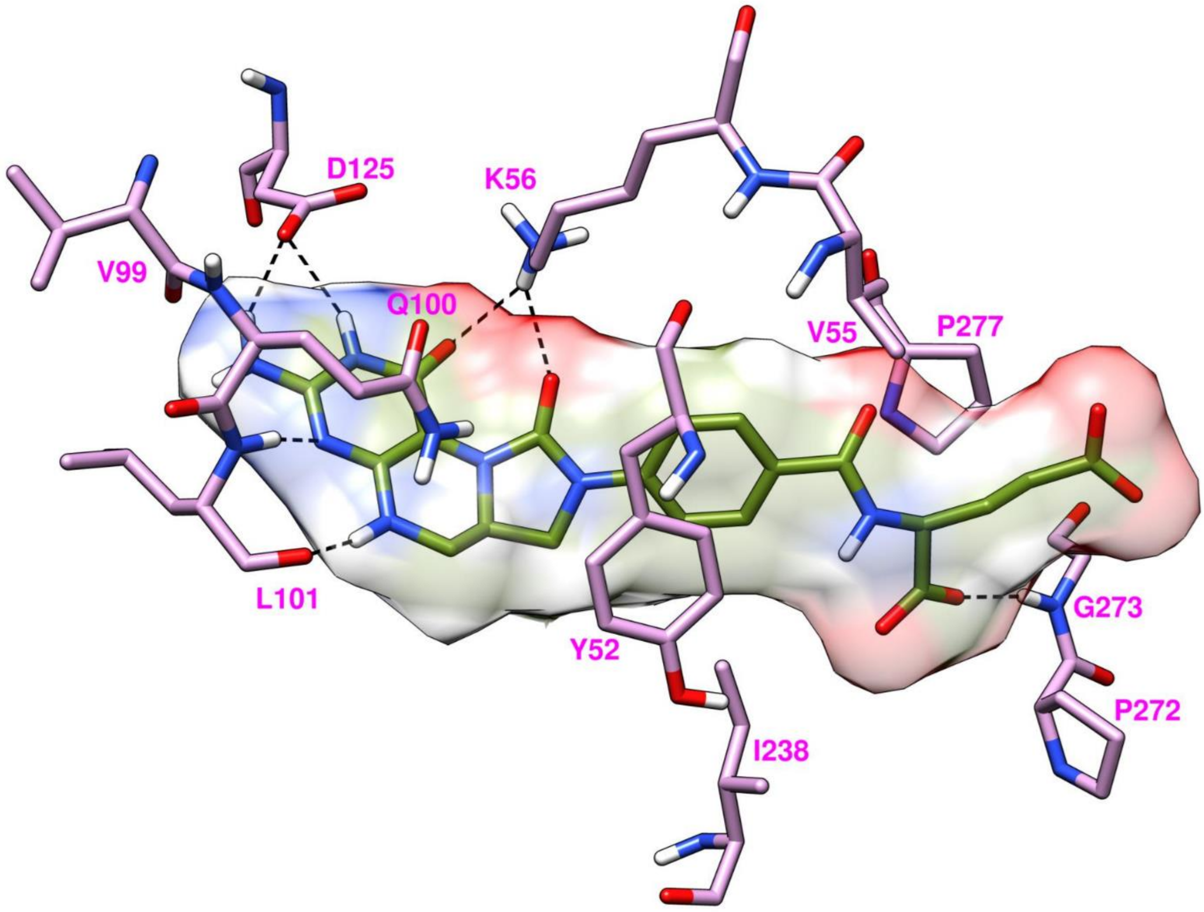

Figure S20. Crystallographic binding mode of the folate-based inhibitor LY345899 in the substrate binding site of MTHFD1 (PDB code: 6ECQ). Inhibitor is colored in olive while the protein residues are colored and labeled in pink.

Table S1. Induced-fit docking results (in kcal/mol) of all compounds in the MTHFD1 allosteric site.

Entry	**Glide score**	**IFD score**
Compound 1	-7.70	-1250.70
Compound 2	-9.25	-1259.37
Compound 3	-9.87	-1253.01
Compound 4R	-10.52	-1255.92
Compound 4S	-10.15	-1255.89
